# The cGAS-STING pathway is a master regulator of OCT4 expression in persistent sarcoma cells and enhances cellular immunotherapy with NK and CIK lymphocytes

**DOI:** 10.1007/s00262-025-04141-w

**Published:** 2025-09-23

**Authors:** Giorgia Giordano, Alessandra Merlini, Federica Capozzi, Giulio Ferrero, Cristina Tucciarello, Shahab Majidi, Simona Comparato, Giulia Mesiano, Elisabetta Liuzzi, Federica Galvagno, Annamaria Massa, Silvia Brusco, Valeria Leuci, Chiara Riganti, Santina Cutrupi, Michele De Bortoli, Lorenzo D’Ambrosio, Dario Sangiolo, Giovanni Grignani, Ymera Pignochino

**Affiliations:** 1 Department of Oncology, Candiolo Cancer Institute, FPO-IRCCS, Candiolo, Turin, Italy; 2 Department of Clinical and Biological Sciences, University of Torino, Turin, Italy; 3 Department of Oncology, University of Torino, Turin, Italy; 4 Medical Oncology, San Luigi Gonzaga University Hospital, Orbassano, Turin, Italy; 5 Italian Institute for Genomic Medicine (IIGM), Turin, Italy; 6 Department of Applied Science and Technology, Politecnico Di Torino, Turin, Italy; 7 Princess Máxima Center for Pediatric Oncology, Utrecht, The Netherlands

**Keywords:** NK/CIK cellular immunotherapy, cGAS-STING-IRF3-IFNβ pathway, Trabectedin, Olaparib, OCT4, Persistent sarcoma cells

## Abstract

Advanced sarcomas have a poor prognosis and limited therapeutic options. Disease recurrence is caused by persistent cells that survive drug treatments. The alkylating agent trabectedin, when combined with the poly (ADP-ribose) polymerase 1 (PARP1) inhibitor olaparib, exhibits variable antitumor effects in advanced sarcomas. In this study, we demonstrate that the expression of the transcription factor OCT4 is upregulated in persistent cells that survive treatment with trabectedin and olaparib, through the cGAS-STING-IRF3-IFNβ pathway. This route also leads to the upregulation of natural killer (NK) and cytokine-induced killer (CIK) lymphocyte activating ligands. These molecular events enhance the antitumor efficacy of immunotherapy with NK and CIK cells, targeting both the bulk population and residual drug-tolerant cells. In conclusion, the activation of the cGAS-STING pathway has a double-edged effect, enriching the OCT4^+^ persistent cell population while increasing the expression of NK/CIK ligands. The addition of olaparib to trabectedin potentiates the cGAS-STING pathway activation and the upregulation of NKG2DLs, while simultaneously counteracting the OCT4 overexpression. Therefore, sequential treatment with trabectedin and olaparib followed by NK/CIK immunotherapy represents a promising strategy against advanced sarcomas and warrants further investigation.

**Supplementary Information:**

The online version contains supplementary material available at 10.1007/s00262-025-04141-w.

## Introduction

Sarcomas are rare, heterogeneous, and malignant tumors of mesenchymal origin. Limited effective therapeutic options are available for the management of advanced, unresectable, or metastatic disease, and their prognosis remains poor [1]. Resistance to cancer treatments is typically attributed to a subset of cells that survive after treatment [2, 3]. The characterization of persistent cells is challenging, and no univocal methods have been established, adding further complexity to the field. Octamer-binding transcription factor 4 (OCT4), encoded by the *POU5F1* gene, is a well-recognized hallmark of pluripotency in both embryonic and quiescent adult cells and plays a key role in cancer [4, 5]. The enrichment of the OCT4^+^ fraction suggests that tumor cells surviving drug treatment acquired stem-like properties, thereby increasing the risk of recurrence [6–8]. Our group previously reported that sarcoma cells spared by conventional treatment are significantly enriched in their OCT4^+^ fraction [9]. Additionally, we demonstrated that the combination of the alkylating agent trabectedin and the poly (ADP-ribose) polymerase-1 (PARP1) inhibitor olaparib exhibits antitumor activity against a subset of sarcomas in both preclinical and clinical studies [10, 11]. Varying degrees of drug synergism were observed, and half of the treated patients did not benefit from the combination. Therefore, we sought to determine whether tumor cells persisting after treatment display characteristics of cancer stemness. Conflicting evidence exists regarding the effect of trabectedin and olaparib on the induction of stem-like traits in tumor cells [12–15]. Moreover, the molecular basis underlying these features in drug-persistent cells is still poorly understood. Trabectedin derivatives, as well as PARP1 inhibitors, trigger cyclic 2′3’GMP-AMP (2′3’-cGAMP) synthetase (cGAS), stimulator of interferon gene (STING), tank-binding kinase I (TBK1), and interferon regulatory factor 3 (IRF3), ultimately leading to interferon (IFN)-β production [16–19]. Moreover, the activation of the cGAS-STING pathway has a dual role, promoting both cancer progression and innate immunity [20–24]. In this context, the immune system’s activity against persistent cells remains far from being fully understood. Adoptive immune cell therapy may represent a promising approach to defeating treatment-resistant cancer cells [25]. The clinical use of innate immune cells such as natural killer (NK) cells and other human leukocyte antigen (HLA)-independent effectors such as cytokine-induced killer (CIK) lymphocytes has demonstrated a favorable clinical safety profile and represents an encouraging therapeutic alternative [26–28]. We previously investigated the activity of immunotherapy with NK and CIK in preclinical in vitro and in vivo models of sarcomas [29], demonstrating their efficacy against the OCT4^+^ fraction [9]. NK and CIK kill their target cells without prior immunization or major histocompatibility complex (MHC) restriction. Several tumor histotypes, including sarcomas, express NK/CIK activating ligands, among which the NKG2D ligands (NKG2DLs), such as the stress-inducible ligands MHC class I chain-related protein A and B (MICA/B) and the UL16 binding proteins (ULBP2/3/5/6) [30–32]. DNA-damaging agents, such as trabectedin, may upregulate the expression of NKG2DLs on cancer cells, potentially enhancing NK/CIK activity [33, 34].

In this work, for the first time, we show that trabectedin-olaparib combination activates the cGAS-STING pathway, leading to a double-edged effect in sarcoma cells: it increases the fraction of OCT4^+^ cells and the expression of NKG2DLs, eventually enhancing NK/CIK antitumor activity against both bulk and OCT4^+^ persistent sarcoma cells. Hence, trabectedin and olaparib show immunomodulatory activity in a preclinical experimental setting, paving the way for sequential immunotherapeutic strategies with NK/CIK-based adoptive cell therapy.

## Materials and methods

### Cell lines, ex vivo expansion of CIK cells, and DMR xenografts

Bone and soft tissue sarcoma primary and commercially available cell lines (chondrosarcoma HT-1080 [35, 36], fibrosarcoma SW684, myxoid/round liposarcoma 402.91, leiomyosarcoma DMR and MES-SA [10], and undifferentiated pleomorphic sarcoma S018 and S006 [29]) were obtained from surgical resection or purchased and cultured as previously described [10, 29]. The NK-92 cell line was purchased from ATCC and cultured in Alpha Minimum Essential Medium with 12.5% horse serum (Gibco) and 12.5% FBS, supplemented with 0.2 mM inositol, 2–0.1 mM 2-mercaptoethanol, 0.02 mM folic acid, 100–200 IU/ml recombinant interleukin (IL)-2 (Chiron Corporation). CIK cells were obtained from the expansion of peripheral blood mononuclear cells (PBMCs) collected as previously described from patients affected by histologically confirmed bone and soft tissue sarcomas [29]. All participants provided written informed consent under a protocol that adheres to the Declaration of Helsinki, and the study was approved by the local Institutional Review Board and Ethics Committee (approval number 296–2020/14-OCT-2020). All the cells were maintained at 37 °C in a humidified atmosphere containing 5% CO_2_. DMR xenograft models were obtained by orthotopic injection of DMR cells into the uterine wall of non-obese diabetic/severe combined immunodeficient (NOD/SCID) mice (Charles River), as previously described [10]. This protocol was approved by the Institutional Animal Welfare Organization (OPBA) of the Candiolo Cancer Institute according to Italian Legislative Decree no. 26 of 03.14.2014 and by the Italian Ministry of Health (Aut. Min. 178/2015-PR).

### Pharmacological treatments

Trabectedin (PharmaMar) and olaparib (Carbosynth) were dissolved in dimethyl sulfoxide (DMSO, Sigma-Aldrich), stored at − 80 °C, and diluted in fresh media immediately before use at a final concentration of 0.5 nM trabectedin and 5 µM olaparib for 24 or 72-h treatment. 2′3’-cGAMP (InvivoGen) was diluted to a final concentration of 10 μM and transfected into cells for 6 or 24 h using Lipofectamine®RNAiMAX (Thermo Fisher Scientific) according to the manufacturer’s protocol. IFNβ (PHC4244, Thermo Fisher Scientific) was stored at − 80 °C and diluted to a final concentration of 10–100–1000 IU/mL in the culture medium for 24-h treatments. Itacitinib (INCB39110, Selleck Chemicals) was stored at − 80 °C and diluted in fresh media immediately before use at a final concentration of 1 μM for 24-h treatments. The IFNβ-neutralizing antibody (AF814, R&D Systems) was stored at − 20 °C and diluted to a final concentration of 0.2 µg/ml for 24-h cell treatment. DMR xenografts were treated with trabectedin and olaparib as previously described [10].

### RNA silencing

Tumor cells were grown to 60% confluence in complete medium and then incubated with 10 nM siRNAs (listed in Supplementary Table S1) and Lipofectamine®RNAiMAX (Thermo Fisher Scientific) diluted in Opti-MEM® (Gibco) without antibiotics, as recommended by the manufacturer. RT-qPCR and western blotting were performed to check silencing efficacy after 24 and 48 h.

### Co-culture with NK-92 or CIK cells, and cell viability assays

After 72 h of trabectedin and olaparib treatment, tumor cells were co-cultured for an additional 72 h with NK or CIK cells at different effector:target ratios. Cell viability was measured by the CellTiter-Glo® Luminescent Cell Viability Assay (Promega) using a GloMax® Discover Microplate Reader (Promega). The killing activity was calculated as the mortality of each effector:target ratio normalized to the mortality in the absence of NK/CIK (effector:target = 0:1). In dedicated experiments, viable and total cell counts were performed by 0.1% Trypan blue dye exclusion (Thermo Fisher Scientific) and OCT4^+^ cells were evaluated by flow cytometry, as described below. Cells were subjected to IRF3 silencing (siIRF3) for 48 h, incubated for 24 h with trabectedin and olaparib as single agents and in combination, and co-cultured for additional 24 h with NK-92 (1:1) or CIK (10:1) cells to test cell viability in comparison with silencer negative control (siCTRL)-treated cells.

### RNA extraction and RT-qPCR

Total RNA was extracted from sarcoma cell lines and xenografts using the Maxwell® RSC miRNA Tissue Kit and the RSC Maxwell Instrument (Promega), following the manufacturer’s instructions. RNA quality and concentration were checked using a DS 11 + spectrophotometer (Denovix Inc). Starting with 500 ng of total RNA, Superscript VILO IV Master Mix (Thermo Fisher Scientific) was used to obtain cDNA. RT-qPCR was performed using TaqMan Fast Advanced Mastermix (Thermo Fisher Scientific) and TaqMan probes (Supplementary Table S2) using the ABI PRISM 7900HT System (Thermo Fisher Scientific). RT-qPCR for IFNAR1/2 and GUSB as housekeeping gene was performed using SYBR Green Master mix (Thermo Fisher Scientific) and an appropriate set of primers (Supplementary Table S3). The expression data were normalized to housekeeping genes, and the fold change for each group of treatments in comparison with untreated controls was calculated using the 2^−ΔΔct^ formula.

### Western blot

Cells were lysed in Lysis Buffer 6 (R&D Systems) to obtain protein extracts, as previously described [37]. Protein concentrations were determined using the BCA Protein Assay (Thermo Fisher Scientific) and Glomax Discover System (Promega). After gel electrophoresis and blotting, nonspecific binding sites were blocked with 10% bovine serum albumin for 1 h. Primary antibodies (listed in Supplementary Table S4) were incubated overnight at 4 °C, followed by 1-h incubation with HRP-conjugated secondary antibodies (Jackson ImmunoResearch Laboratories). Imaging and quantification were performed using the Bio-Rad Chemidoc™ Touch Imaging System (Bio-Rad Laboratories), and intensity was quantified using QuantityOne (Bio-Rad Laboratories), using vinculin or β-actin as a normalizer.

### Flow cytometry

Tumor cells were incubated for one hour at + 4 °C in the dark with fluorochrome-conjugated primary antibody against OCT4 after permeabilization with a fix-and-perm kit (Thermo Fisher), or directly with NKG2DL antibodies (Supplementary Table S5) The fluorescent signal was detected by ADP Cyan (Beckman Coulter Srl) and analyzed using Summit v4.3 software (Beckman Coulter Srl). Gating strategies are shown in Supplementary Figure S1. NK and CIK cell phenotypes were evaluated using fluorochrome-conjugated monoclonal antibodies against CD3, CD8, CD56, CD134, and CD226 (Supplementary Table S5).

### RNA sequencing and bioinformatic analysis

After RNA extraction, RNA concentration was determined using the Qubit RNA BR (broad range) assay kit (Thermo Fisher Scientific) and a Qubit® 3.0 Fluorometer (Thermo Fisher Scientific). RNA fragmentation was assessed using the 2100 Bioanalyzer High-Sensitivity RNA Assay Kit (Agilent Technologies). The total RNA was processed for RNA-sequencing analysis with TruSeq Stranded Total RNA Gold (Illumina) following the manufacturer’s instructions and sequenced on a NovaSeq6000 (Illumina). Sequencing reads were preprocessed and filtered using fastp software v0.23.1 [38]. Filtered reads were aligned to transcript annotations using Salmon v1.4.0 in default settings with seqBias and gcBias options to correct hexamer priming and GC-content biases, respectively [39]. Gencode v36 was used as a reference transcriptome. Read alignments were preprocessed with the tximport v1.18.0 R package [40] and expression levels were normalized to transcripts per million (TPM). DESeq2 v1.30.1 [41] was used to perform the differential expression analysis. A gene was considered differentially expressed if it was associated with an adjusted p-value < 0.05, and a median TPM > 1 in all samples. Metascape v3.5 [42] was used in the default settings for functional enrichment analysis. Raw sequencing data were deposited in Gene Expression Omnibus (GEO) with the identifier GSE239639.

### Immunofluorescence

Cytosolic dsDNA was visualized in drug-treated cells and formalin-fixed paraffin-embedded (FFPE) DMR xenografts. Cells were seeded in an 8-well chamber (Ibidi), treated with trabectedin, olaparib, or their combination for 24 h and fixed with cold 4% paraformaldehyde (#sc-281692; Santa Cruz Biotechnology) for 10 min at room temperature. FFPE-DMR xenografts were 2-µm sectioned using an RM 2125 microtome (Leica Biosystems), mounted on polarized glass slides (Leica Biosystem), and dried at 40 °C for 30 min. Sections were deparaffinized, subjected to antigen retrieval with sodium citrate buffer pH 6 (#C9999, Sigma Aldrich) at 96 °C for 30 min, and permeabilized in 0.01% Triton-X100, 0.1% Tween-20, and phosphate-buffer saline (PBST) for 10 min. Nonspecific binding sites were blocked for 30 min in blocking buffer (1% BSA, 2.25% Glycine, PBST). Finally, the sections were incubated overnight at 4 °C with dsDNA antibody (Supplementary Table S4), followed by a 1-h incubation with Alexa Fluor 488 secondary antibody (Thermo Fisher Scientific), DAPI (Thermo Fisher Scientific), and phalloidin (PHN-Alexa 647, Sigma Aldrich). Imaging was performed using an SPE II confocal microscope (Leica) with an oil immersion objective (63x). The number of intracellular dsDNA spots was quantified using ImageJ software as the total occupied area within the cell.

### Spheroid-formation assay

HT-1080 cells were seeded in ultra-low-attachment round-bottom 96-well plates (Corning) at a density of 1000 viable cells/well in DMEM-F12 (Sigma Aldrich), B27 (Gibco), 10 ng/mL h-EGF, 10 ng/mL h-FGF, 4 μg/mL insulin (Thermo Fisher Scientific) and penicillin/streptomycin, with or without 2′3’-cGAMP (10 µM), centrifuged at 1000 rpm for 10 min, and cultured for 14 days, replacing half of the medium every 72 h. Spheroid-forming ability was monitored by taking photographs at different time points (1, 3, 6, 10, and 14 days) using an inverted phase-contrast microscope at 10 × magnification (Leica). Digital images of the cultured spheroids were analyzed using ImageJ software [43] to calculate their area (μm^2^). The number of viable cells was counted by trypan blue dye exclusion, and spheroid growth was quantified at the end of the experiment (day 14) using the Cell Titer-Glo® assay.

### Crystal violet growth assay

HT-1080 cells were seeded in 12-well plates in complete medium and, after 24 h treated with 2′3’-cGAMP for 7 days, replacing the medium every 72 h. Alternatively, cells were treated with trabectedin, olaparib, or their combination for 72 h and then after medium replacement, surviving cells were cultured for another 72 h in the presence or absence of NK-92 (effector:target ratio = 1:12) or CIK (effector:target ratio = 1:1) cells. At the end of the experiments, the cells were stained with 0.1% crystal violet (Sigma-Aldrich), and images were captured using the high-throughput imaging platform LIPSI.

### ELISA assay

Cell supernatants were collected and analyzed using the Human IFNβ ELISA Kit (DIFNB0, R&D Systems) according to the manufacturer’s instructions. Absorbance was measured at 450 nM, with wavelength correction at 570 nM, using the Glomax Discover System (Promega).

### Chromatin immunoprecipitation (ChIP)

ChIP was conducted using the Zymo-Spin ChIP kit (D5210, Zymo Research), following the manufacturer’s instructions. Briefly, 10^6^ cells were used per 1 ml of ChIP reaction, and sonication was conducted using Bioruptor Pico (Diagenode) for five cycles, with 30 s ON and 30 s OFF at 40% amplitude. For each ChIP reaction, 2.5 µg of antibody (listed in Supplementary Table S4) was added. qPCR analysis was performed with immunoprecipitated samples or input controls in each reaction mixed with SYBR Green Master mix (Thermo Fisher Scientific) and an appropriate set of primers designed for the promoter region of *POUF5F1* and *IFNB1* (Supplementary Table S6). Briefly, the genomic region ranges from − 2000 to + 400 bp of the *POU5F1* gene. The Transcription Start Site (TSS) was explored for candidate IRF3 binding motifs using the Eukaryotic Promoter Database (EPD), Expasy [44], and Multiple Em for Motif Elicitation (MEME) tool [45], considering the positional weight matrices annotated in JASPAR CORE 2024 [46]. Specifically, IRF3 binding motifs annotated in this database were analyzed, and only those associated with a *p*-value < 0.05 were considered statistically significant. The IRF3 consensus sequences were identified at − 1598 bp (*p* < 0.001) and at − 91 bp (*p* < 0.001) from the TSS in the *POU5F1* and *IFNB1* promoter regions, respectively (Supplementary Figure S2).

### Study design and Statistical analysis

No prior sample-size calculations were performed. RT-qPCR of *POU5F1*, cGAS-STING pathway key transducers, and NKG2DL expression were conducted with 7 cell lines and one xenograft model. Protein expression and in vitro experiments were performed with 3 cell lines, and validation of specific molecular mechanisms was conducted with one cell line, as specified in the results section. No inclusion or exclusion criteria were applied. No randomization, blinding, or confounding controls were applied. Differences between treatment groups (including untreated controls) were analyzed with two-way ANOVA (Tukey’s test with Bonferroni’s correction for multiple comparisons) using Prism 8 GraphPad Software. The results are reported as mean ± standard deviation of at least three replicates. No replicate exclusions were made. Results with a *p*-value < 0.05 were deemed to be statistically significant.

## Results

### Persistent sarcoma cells spared by the trabectedin-olaparib combination express OCT4 in vitro and in vivo

**Fig. 1:**
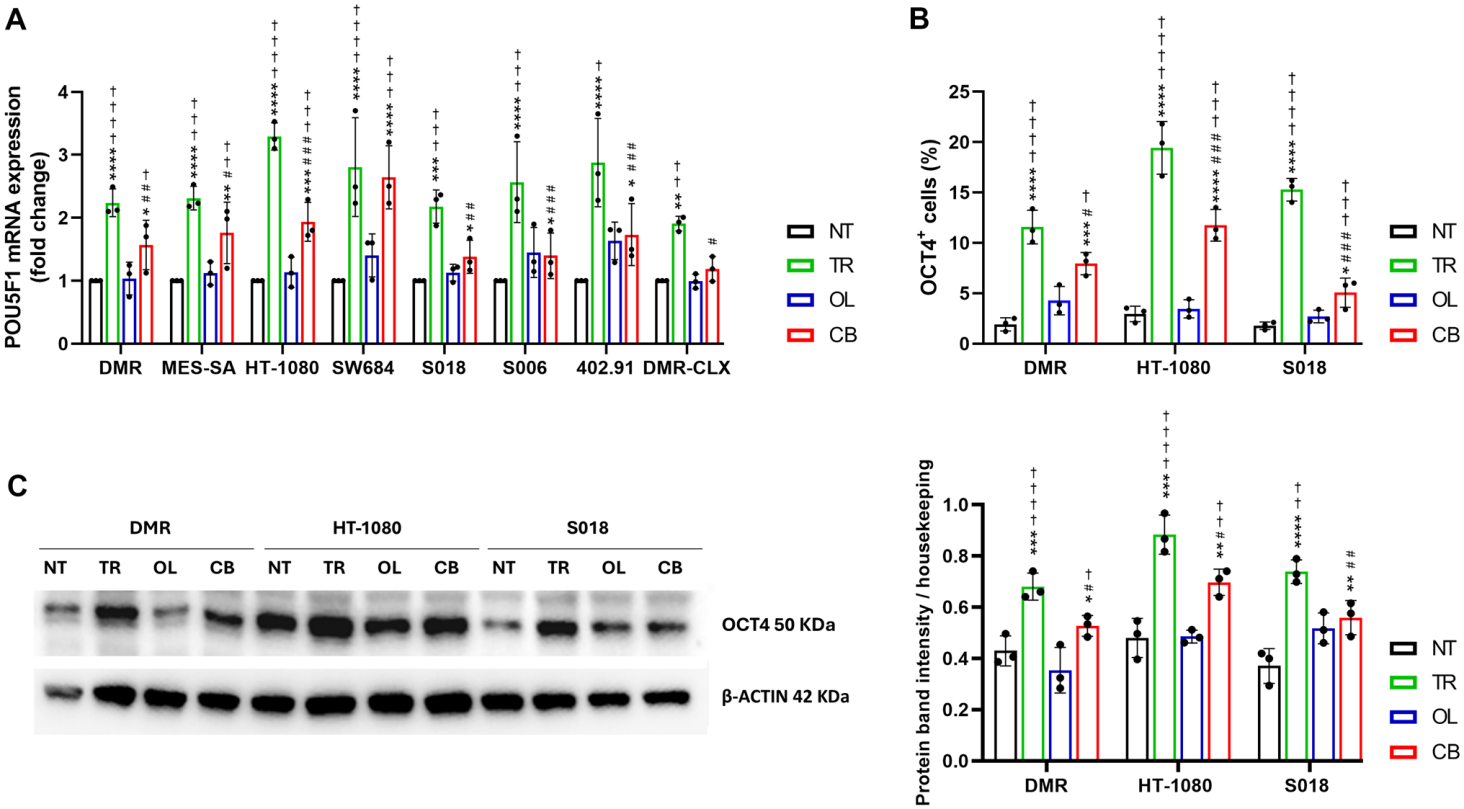
Expression of OCT4 in persistent sarcoma cells spared by trabectedin and olaparib treatments. **A** Real-time PCR analysis of *POU5F1* mRNA expression level (fold change) in seven sarcoma cell lines, and xenografts (DMR-CLX) after 72-h or 21-day drug treatment, respectively. **B** Flow cytometry analysis of OCT4^+^ cells in DMR, HT-1080, and S018 cells after 72-h drug treatment. **C** Representative western blot analyses and protein band intensity quantification of OCT4 and β-actin after 72 h of drug treatment. NT = untreated, TR = trabectedin-treated, OL = olaparib-treated, CB = combination-treated models. Data are shown as mean ± standard deviation (n = 3; * *p* < 0.01, ** *p* < 0.01, *** *p* < 0.001, **** *p* < 0.0001 vs. untreated; # *p* < 0.05, ## *p* < 0.01, ### *p* < 0.001, #### *p* < 0.0001 vs. trabectedin-treated; † *p* < 0.05, †† *p* < 0.01, ††† *p* < 0.001, †††† *p* < 0.0001 vs. olaparib-treated models)

Since previous studies reported an increase in OCT4^+^ cells following targeted and chemotherapeutic treatments [9], we investigated OCT4 expression in persistent sarcoma cells spared by trabectedin and olaparib. In 7 tested sarcoma cell lines, *POU5F1* mRNA expression levels were significantly higher in cells surviving trabectedin compared to untreated controls, olaparib, and combination treatments. However, the combination treatment significantly increased *POU5F1* mRNA expression compared to that in the untreated control (Fig. 1A). In *vivo*, DMR xenografts that survived trabectedin treatment exhibited higher levels of *POU5F1* mRNA compared to the other treatment groups (Fig. 1A). Furthermore, we confirmed the modulation of OCT4 expression at the protein level by flow cytometry, showing that trabectedin as a single agent significantly increased the percentage of OCT4^+^ cells in three out of 3 cell lines compared to untreated controls. Olaparib, as single agent, did not modify the OCT4^+^ population fraction. Furthermore, in cells treated with the combination, the percentage of OCT4^+^ cells was significantly higher than that in untreated cells, but lower than that in trabectedin-treated cells (Fig. 1B). In addition, western blot analysis on OCT4 expression confirmed these results (Fig. 1C).

### Trabectedin-olaparib combination upregulates the genotoxicity pathway and inhibits the cellular response to DNA damage

**Fig. 2:**
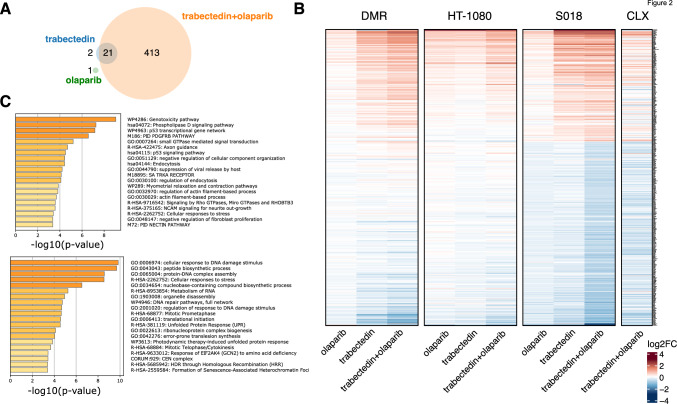
Transcriptionally modulated genes and pathways in olaparib, trabectedin, and combination-treated sarcoma models. **A** Venn diagram showing genes differentially expressed in trabectedin, olaparib, and combination-treated cells in comparison with untreated ones. **B** Heatmap of the differentially expressed genes in DMR, HT-1080, and S018 cells, and DMR xenografts (CLX) after 72-h and 21-day drug treatment, respectively. **C** Functional enriched terms within the list of upregulated (top) and downregulated (bottom) genes

The transcriptomic profiles of persistent sarcoma cells treated with trabectedin and olaparib were analyzed in cell lines and xenograft models. Total RNA sequencing revealed that 437 genes were differentially expressed between treated *versus* untreated cells (adj. *p* < 0.05, median TPM > 1) (Fig. 2A). Specifically, trabectedin, olaparib, and their combination significantly modulated 23, 1, and 434 genes, respectively. Notably, 21 genes were modulated by both trabectedin and its combination, whereas 413 genes were significantly modulated only by the combination (Fig. 2A). S018 cells showed the most relevant gene expression variation between untreated and combination-treated samples, accounting for 347 differentially expressed genes (adj. *p* < 0.05, Supplementary Table S7). As shown in Fig. 2B, the fold-change in expression was consistent among DMR, HT-1080, and S018, with a prevalence of gene downregulation. These expression patterns were also observed in vivo (Fig. 2B). Functional enrichment analysis of both cell lines and xenografts showed that the *Genotoxicity pathway (WP4286)* was the most enriched term for upregulated genes, and the *Cellular response to DNA damage stimulus (GO:0006974)* was the most downregulated (Fig. 2C and Supplementary Table S8). Volcano plot showed the differentially expressed genes in the three cell lines after drug treatments and in DMR xenografts (Supplementary Figure S3).

### Trabectedin-olaparib combination increases cytosolic dsDNA and activates the cGAS-STING-IRF3-IFNβ pathway

**Fig. 3:**
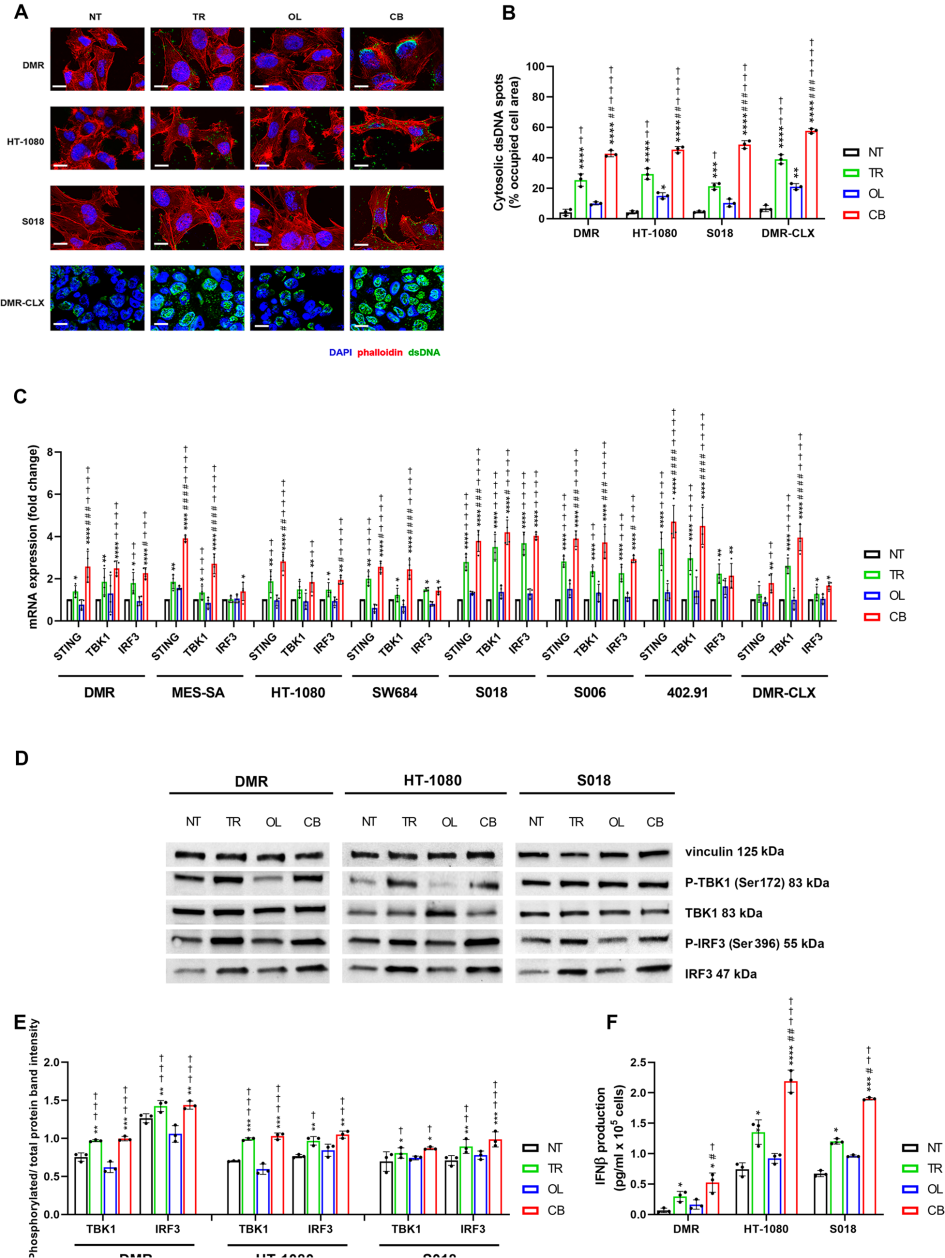
Activation of cGAS-STING cytosolic dsDNA sensing pathway in trabectedin, olaparib, and combination-treated sarcoma models. **A** Representative immunofluorescence of cytosolic dsDNA in DMR, HT-1080, and S018 cells, and DMR xenografts after 24-h and 21-day drug treatment, respectively. Scale bar = 10 µm (cell lines) and 20 µm (DMR-CLX). **B** Quantification of occupied cell area by cytosolic dsDNA spots. **C** Real-time PCR analysis of STING, TBK1, and IRF3 mRNA expression levels (fold change vs. NT) in cell lines and xenografts after 72-h and 21-day drug treatment, respectively (n = 4). **D** Representative western blot analyses of P-TBK1 (Ser172), TBK1, P-IRF3 (Ser396), IRF-3, and vinculin after 72-h drug treatment. **E** Quantification of phosphorylated and total protein band intensity ratio normalized to vinculin as housekeeping. **F** ELISA of IFNβ production in DMR, HT-1080, and S018 cells after 72-h drug treatment. NT = untreated, TR = trabectedin-treated, OL = olaparib-treated, CB = combination-treated models. Data are shown as mean ± standard deviation (n = 3; * *p* < 0.01, ** *p* < 0.01, *** *p* < 0.001, **** *p* < 0.0001 vs. untreated; # *p* < 0.05, ## *p* < 0.01, ### *p* < 0.001, #### *p* < 0.0001 vs. trabectedin; † *p* < 0.05, †† *p* < 0.01, ††† *p* < 0.001, †††† *p* < 0.0001 vs. olaparib)

Genotoxic stress activation combined with inhibition of the DNA damage response can lead to the leakage of double-stranded DNA (dsDNA) into the cytosol. Consistent with this, confocal microscopy revealed a marked accumulation of cytosolic dsDNA 24 h after treatment. Notably, the combination treatment induced significantly greater dsDNA accumulation compared to either single agents or controls, as further confirmed in vivo in DMR xenografts (Fig. 3A–B). The accumulation of cytosolic dsDNA is known to activate the cGAS–STING signaling pathway [47]. Thus, as the first step, we analyzed the expression of the key transducers (STING, TBK1, and IRF3) in all seven cell lines and DMR xenografts. The trabectedin-olaparib combination resulted in a significant upregulation of these genes compared to untreated controls and showed a variable modulation compared to single agents (Fig. 3C). To further assess activation of the cGAS–STING pathway, we performed western blot analysis of phosphorylated TBK1 (Ser172) and IRF3 (Ser396), along with their corresponding total protein levels, in DMR, HT-1080, and S018 cells. A significant increase in TBK1 and IRF3 phosphorylation was observed in cells treated with trabectedin or the combination, but not with olaparib alone, compared to untreated controls. No appreciable differences were detected between trabectedin monotherapy and the combination treatment (Fig. 3D–E). Moreover, the production of IFNβ encoded by the *IFNB1* gene, as the downstream effector of cGAS–STING pathway, was significantly increased following trabectedin and combination treatment compared to untreated controls and was considerably higher in the combination compared to single agents (Fig. 3F).

### The cGAS-STING-IRF3-IFNβ pathway controls *POU5F1* gene expression

**Fig. 4:**
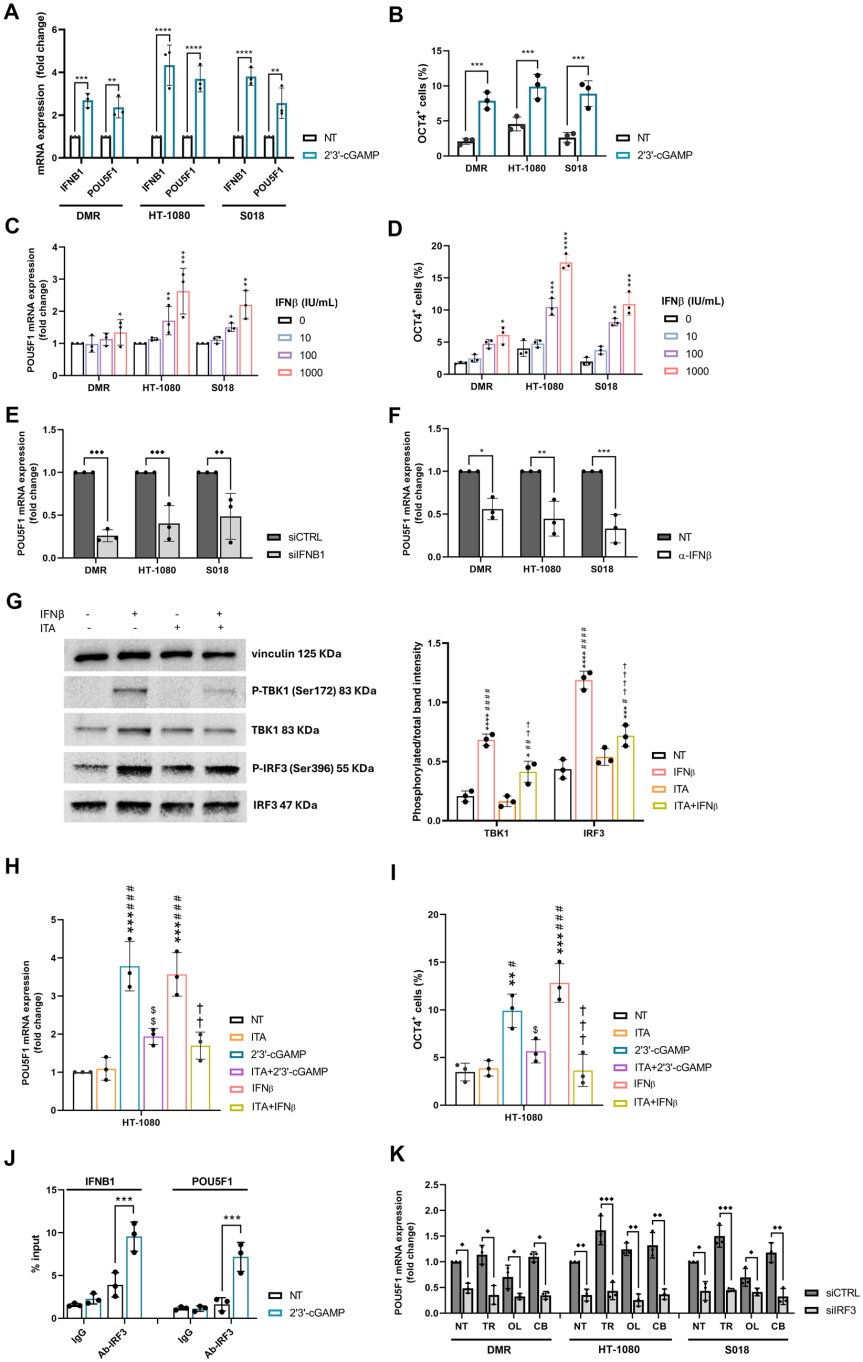
cGAS-STING-IFNβ pathway controls OCT4 expression**. A** Real-time PCR analysis of *IFNB1* and *POU5F1* mRNA expression level (fold change) after 6 h of 2′3’-cGAMP treatment. **B** Flow cytometry analysis of OCT4^+^ cells after 24 h of 2′3’-cGAMP treatment. **C–D** Real-time PCR of *POU5F1* mRNA expression level (fold change) and flow cytometry analyses of OCT4^+^ cells after 24 h of IFNβ treatments.** E** Real-time PCR analysis of *POU5F1* mRNA expression level (fold change *vs.* siCTRL) in siCTRL and si*IFNB1*-transfected cells. **F** Real-time PCR analysis of *POU5F1* mRNA expression level (fold change) after α-IFNβ neutralizing antibody. **G** Western blot analysis and protein band intensity quantification of P-TBK1(Ser172), TBK1, P-IRF3(Ser396), IRF3 and vinculin after IFNβ treatment, as single agents or in combination with itacitinib. **H–I** Real-time PCR of *POU5F1* mRNA expression (fold change) and flow cytometry analyses of OCT4 in cells treated with 2′3’-cGAMP or IFNβ, as single agents or in combination with itacitinib. **J** q-PCR of *IFNB1* and *POU5F1* promoter regions following ChIP with IRF3 antibody in HT-1080 cells treated with 2′3'-cGAMP. **K** Real-time PCR analyses of *POU5F1* mRNA expression level (fold change *vs.* siCTRL NT) in siCTRL and siIRF3-transfected cells after 24-h drug treatment. siCTRL = control siRNA-transfected cells, si*IFNB1* = *IFNB1*-silenced cells, siIRF3 = IRF3-silenced cells, NT = untreated, TR = trabectedin-treated, OL = olaparib-treated, CB = combination-treated cells, ITA = itacitinib. Data are shown as mean ± standard deviation (n = 3, *p < 0.05, ** *p* < 0.01, *** *p* < 0.001, **** *p* < 0.0001 *vs.* untreated; # *p* < 0.05, ## *p* < 0.01, ### *p* < 0.001, #### *p* < 0.0001 *vs.* ITA; †† *p* < 0.01, †††† *p* < 0.0001 *vs.* IFNβ; $$ *p* < 0.01, $$$ *p* < 0.001 vs 2′3'-cGAMP; ◆ *p* < 0.05, ◆◆ *p* < 0.01, ◆◆◆ *p* < 0.001 ◆◆◆◆ *p* < 0.0001 *vs*. siCRTL)

To understand whether cGAS-STING pathway activation plays a role in persistent cells spared by drug treatment, we investigated its effect on *POU5F1* expression. Treatment with 2′3’-cGAMP induced a significant upregulation of both *IFNB1* as a positive control and *POU5F1* mRNA expression levels (Fig. 4A). The increment of OCT4 protein expression after 2′3’-cGAMP treatment was also confirmed by flow cytometry (Fig. 4B). The functional readout of this upregulation was investigated using sphere-forming and growth assays in the presence or absence of 2′3’-cGAMP. DMR and S018 cells were unable to grow in sphere-forming conditions (data not shown), whereas HT-1080 cells showed a significant increase in growth as spheroids when treated with 2′3’-cGAMP, compared to untreated controls (Supplementary Figure S4A–C). To confirm this evidence, we tested the colony growth ability of HT-1080 cells treated with 2′3’-cGAMP, showing a significantly higher number of colonies compared to untreated controls (Supplementary Figure S4D).

In addition, after verifying the expression of IFNβ receptors (IFNAR1/2) in sarcoma cells (Supplementary Figure S5A-B), we directly treated sarcoma cells with IFNβ, showing a dose-dependent OCT4 upregulation at both the mRNA and protein levels (Fig. 4C–D). To further validate the role of IFNβ in the control of OCT4 expression, we inhibited its production by silencing *IFNB1* gene expression in three cell lines (Supplementary Figure S5C), showing a significant decrease in *POU5F1* mRNA expression in IFNβ-silenced cells compared to siCTRL cells (Fig. 4E). Moreover, we treated the 3 cell lines with an IFNβ-neutralizing antibody, observing a significant reduction of *POU5F1* mRNA expression, compared to the untreated control (Fig. 4F). In addition, we investigated the role of the IFNβ downstream pathway in the control of OCT4 expression. Treatment with IFNβ induced the phosphorylation of TBK1 (S172) and IRF3 (S396) (Fig. 4G), suggesting a feedforward loop. Moreover, by using the JAK1 inhibitor itacitinib, which inhibited the 2′3’-cGAMP and IFNβ-induced STAT1 phosphorylation (Y701) as expected (Supplementary Figure S5D-E), we observed the reduction of the IFNβ-induced TBK1 and IRF3 phosphorylation (Fig. 4G). Consequently, the 2′3’-cGAMP and IFNβ-induced *POU5F1* mRNA and protein upregulation were significantly reduced by adding itacitinib (Fig. 4H–I).

To further elucidate the regulation of OCT4 expression, we explored whether *POU5F1* gene transcription could be directly activated by the transcription factor IRF3. ChIP assay showed that treatment with 2′3’-cGAMP significantly induced binding of IRF3 to the promoter regions of *POU5F1* as well as *IFNB1,* used as positive control (Fig. 4J)**.** To validate the role of IRF3 in the control of OCT4 expression after drug treatment, we silenced *IRF3* gene expression in three cell lines. The siIRF3 significantly reduced IRF3 mRNA expression under all treatment conditions (Supplementary S5F). Notably, *POU5F1* mRNA expression in *IRF3*-silenced cells was significantly reduced compared with siCTRL-treated cells. Moreover, silencing of *IRF3* also reduced *POU5F1* expression in drug-treated cells (trabectedin, olaparib, and combination) (Fig. 4K).

**Fig. 5:**
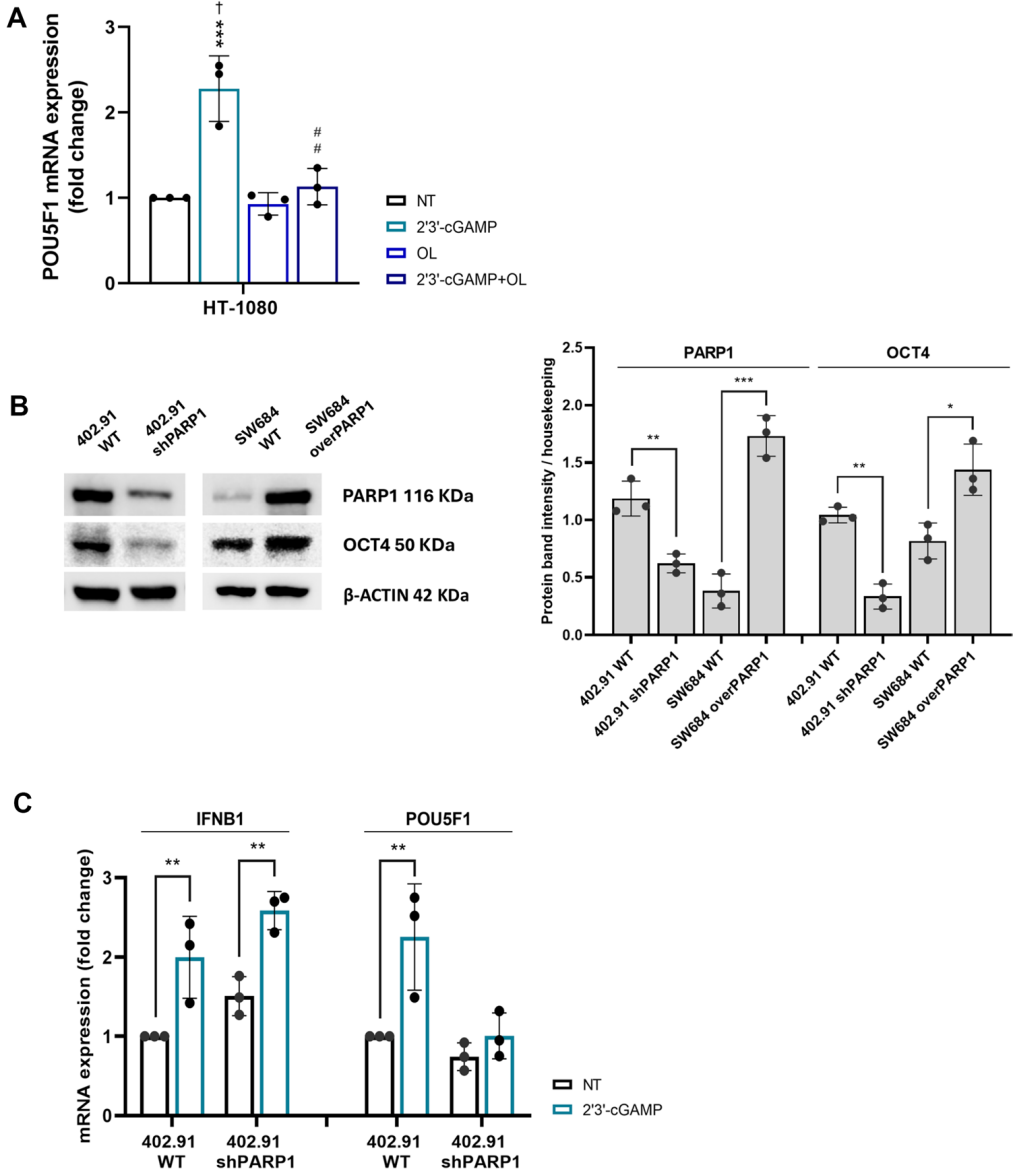
PARP1 is involved in IFNβ-mediated OCT4 regulation. **A** Real-time PCR analyses of *POU5F1* mRNA expression level (fold change *vs* untreated) after the treatment with 2′3’-cGAMP, and olaparib, as single agents or in combination, in HT-1080 cell lines. **B** Representative western blot analysis and protein band intensity quantification of OCT4 and β-ACTIN in 402.91 WT, 402.91 shPARP1, SW684 WT, and SW684 overPARP1 cell lines. **C** Real-time PCR analyses of *POU5F1* mRNA expression level (fold change *vs* untreated) after the treatment with 2′3’-cGAMP in 402.91 wild type and stably PARP1-silenced cell lines. NT = untreated, OL = olaparib. WT = wild type. shPARP1 = PARP1-silenced; overPARP1 = PARP1-overexpressing. (n = 3, *p < 0.05, ** *p* < 0.01, *** *p* < 0.001, **** *p* < 0.0001 *vs.* untreated; ††† *p* < 0.001, *vs.* olaparib-treated cells; ## *p* < 0.01 *vs.*2′3’-cGAMP)

To explain why OCT4 upregulation in combination-treated cells was significantly lower than that in trabectedin-treated cells, even though cGAS-STING was activated, we assessed OCT4 expression in the presence of 2′3’-cGAMP and olaparib. Notably, PARP1 inhibition counteracted 2′3’-cGAMP-induced *POU5F1* mRNA upregulation (Fig. 5A), evoking the effects observed in trabectedin-olaparib combination-treated cells. To further validate these findings, we measured OCT4 expression after PARP1-silencing and overexpression. Stably PARP1-silenced (shPARP1) 402.91 and PARP1-overexpressing (overPARP1) SW684 cells displayed a lower and higher OCT4 expression, respectively, if compared to their parental counterpart (Fig. 5B). In addition, the treatment with 2′3’-cGAMP induced a significant mRNA upregulation of *IFNB1*, but not of *POU5F1*, in 402.91 shPARP1 cells (Fig. 5C).

### The cGAS-STING-IRF3-IFNβ pathway activation increases the NKG2DLs

**Fig. 6:**
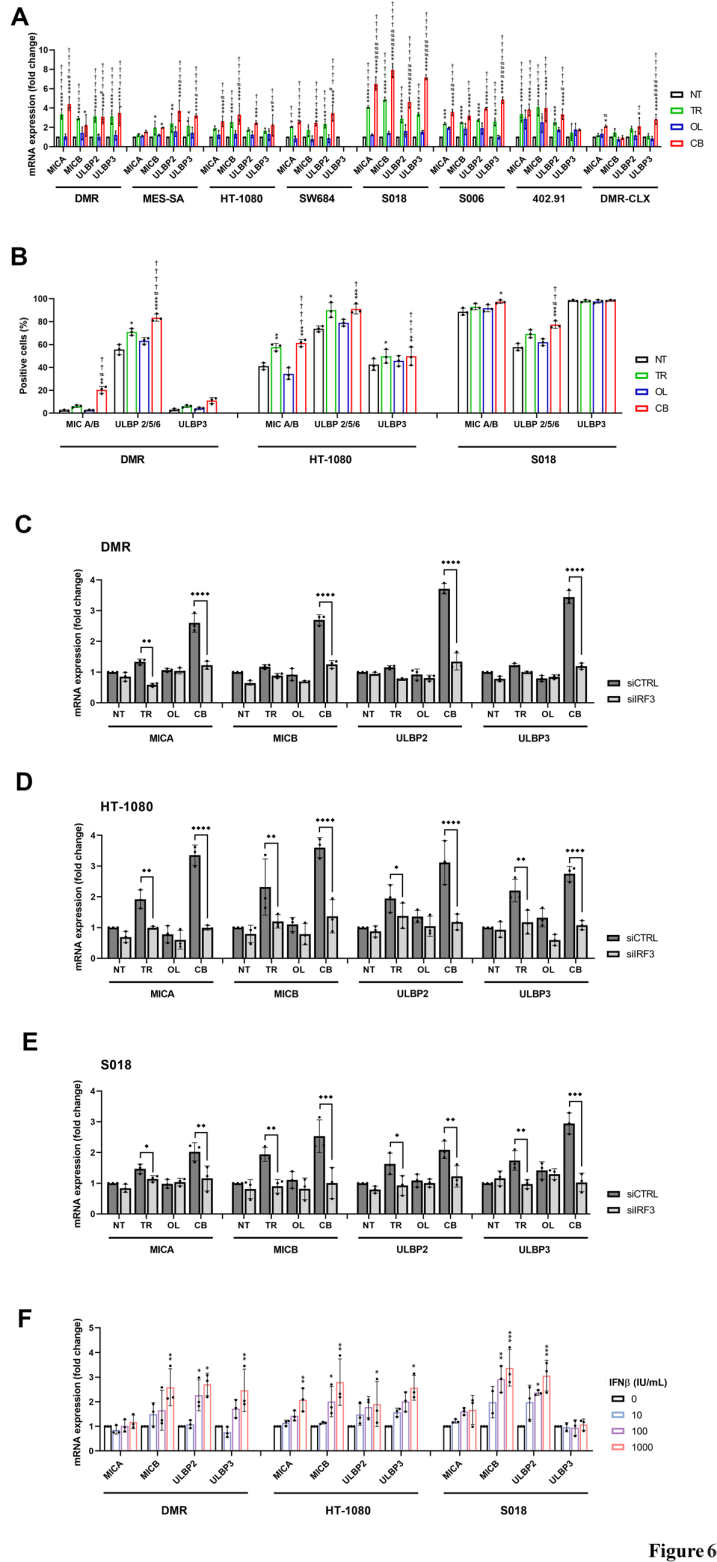
cGAS-STING-IRF3-IFNβ pathway controls NKG2DL expression. **A** Real-time PCR analyses of NKG2DL mRNA expression (fold change *vs.* NT) in seven sarcoma cell lines and DMR xenografts (DMR-CLX) after 72-h and 21-day drug treatments, respectively. **B** Flow cytometry analyses of NKG2DL protein expression on the plasma membrane of sarcoma cell lines after 72 h of drug treatment. **C–E** Real-time PCR analysis of NKG2DL mRNA expression level (fold change *vs.* siCTRL NT) in siCTRL and siIRF3-transfected cells after 24-h drug treatment in DMR, HT-1080, and S018 cell lines, respectively. **F** Real-time PCR analysis of NKG2DL mRNA expression level (fold change *vs.* NT) after 24-h IFNβ treatment. siCTRL = control siRNA-transfected cells, siIRF3 = IRF3-silenced cells, NT = untreated, TR = trabectedin, OL = olaparib, CB = combination. Data are shown as mean ± standard deviation (n = 3; * *p* < 0.05, ** *p* < 0.01, *** *p* < 0.001, **** *p* < 0.0001 *vs.* untreated; # *p* < 0.05, ## *p* < 0.01, ### *p* < 0.001, #### *p* < 0.0001 *vs.* trabectedin; † *p* < 0.05, †† *p* < 0.01, ††† *p* < 0.001, †††† *p* < 0.0001 *vs.* olaparib; ◆ *p* < 0.05, ◆◆ *p* < 0.01, ◆◆◆ *p* < 0.001 ◆◆◆◆ *p* < 0.0001 *vs*. siCRTL)

Since previous findings showed that the cGAS–STING pathway upregulates NK/CIK-activating ligand expression [21, 48, 49], we investigated whether treatment with the trabectedin–olaparib combination induces a similar effect. The mRNA expression of MICA/B and ULBPs was upregulated by the combination in all seven sarcoma cell lines, compared to untreated controls and single agents (Fig. 6A). Similar results were obtained in vivo in combination-treated DMR xenografts (Fig. 6A). Moreover, the NKG2DL modulation was validated at the protein level in three cell lines (Fig. 6B).

To confirm the involvement of the cGAS–STING pathway in drug-induced NKG2DL upregulation, we inhibited its activation by silencing IRF3, a key downstream effect. In IRF3-silenced cells, the drug-induced IFNβ production (Supplementary Figure S6A) and the NKG2DL mRNA upregulation were impaired (Fig. 6C–E). These findings were also validated at the protein level in HT-1080 cells, where IRF3 silencing significantly impaired the NKG2DL upregulation induced by trabectedin and combination treatments (Supplementary Figure S6B). In addition, the direct treatment with IFNβ significantly increased the NKG2DL mRNA expression, compared to untreated controls (Fig. 6F).

### NK/CIK lymphocytes exert antitumor activity against both bulk and OCT4^+^ cell fractions

**Fig. 7:**
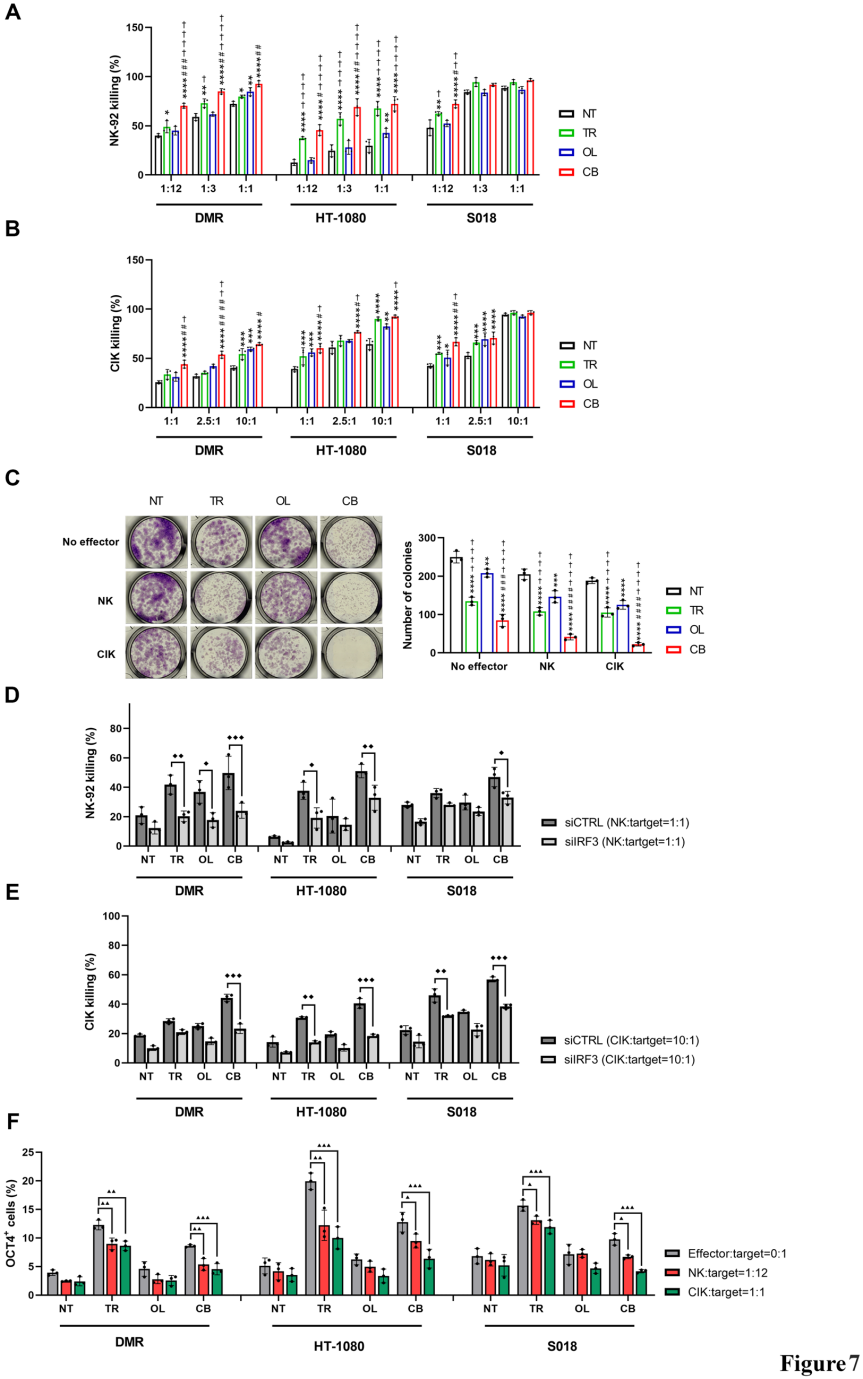
NK/CIK antitumor activity against persistent sarcoma cells. **A–B** Killing activity of NK-92 (1:12, 1:3, 1:1 effector:target ratio) and CIK (1:1, 2.5:1, 10:1 effector:target ratio) against DMR, HT-1080, and S018 cells after 72-h pretreatment with trabectedin and olaparib as a single agent and in combination. **C** Representative crystal violet growth assay of HT-1080 cells, after 72-h drug pretreatment, co-cultured with NK-92 (1:12 effector:target ratio), or CIK (1:1 effector:target ratio) and relative quantification. **D–E** Killing activity of NK-92 (1:1 effector:target ratio) or CIK (10:1 effector:target ratio) after 24-h drug pretreatment of IRF3-silenced cells. **F** Flow cytometry analysis of OCT4^+^ cells after 72-h drug pretreatment in sarcoma cells co-cultured with NK-92 (1:12 effector:target ratio) or CIK (1:1 effector:target ratio). siCTRL = control siRNA-transfected, siIRF3 = IRF3-silenced, NT = untreated, TR = trabectedin-pretreated, OL = olaparib-pretreated, CB = combination-pretreated cells. Data are shown as mean ± standard deviation (n = 3; *p < 0.05, ** *p* < 0.01, *** *p* < 0.001, **** *p* < 0.0001 *vs.* untreated; # *p* < 0.05, ## *p* < 0.01, ### *p* < 0.001 *vs.* trabectedin; † *p* < 0.05, †† *p* < 0.01, ††† *p* < 0.001, †††† *p* < 0.0001 *vs.* olaparib ◆ *p* < 0.05, ◆◆ *p* < 0.01, ◆◆◆ *p* < 0.001, ◆◆◆◆ *p* < 0.0001 *vs*. siCRTL; ▲ *p* < 0.05, ▲▲ *p* < 0.01, ▲▲▲ *p* < 0.001, ▲▲▲▲ *p* < 0.0001 *vs.* 0:1 effector:target ratio)

Since activation of the cGAS–STING pathway enhanced NKG2DL expression in sarcoma cells following trabectedin–olaparib treatment, we next evaluated the antitumor activity of NK/CIK cells after drug exposure. Notably, NK-92 and CIK cells (immunophenotype detailed in Supplementary Figure S7) exhibited robust killing activity in three out of three cell lines, at the lowest effector:target ratio (1:12 for NK and 1:1 for CIK) after trabectedin-olaparib combination pretreatment, compared to the other pretreatment conditions (Fig. 7A-B). This potentiation was further validated using crystal violet staining. Both NK and CIK cells significantly decreased tumor cell growth after pretreatment with the combination compared to the other pretreatment conditions (Fig. 7C). Next, we functionally validated the role of the cGAS-STING pathway in boosting the NK/CIK-killing activity. Blocking cGAS-STING activation by *IRF3* silencing significantly reduced the antitumor activity of sequential treatment with trabectedin-olaparib combination followed by NK or CIK cells (Fig. 7D–E). Finally, we investigated whether the OCT4^+^ population, enriched after drug treatment, might be killed by NK and CIK cells. We observed that both NK-92 (effector-target ratio 1:12) and CIK (effector-target ratio 1:1) significantly decreased the OCT4^+^ fraction in trabectedin and combination-pretreated cells (Fig. 7F).

## Discussion

Conventional therapies, including chemotherapy, radiotherapy, and targeted agents, are effective against the bulk of tumor cells; however, they fail to eradicate subpopulations endowed with cancer stemness features, which are key drivers of disease relapse and metastatic progression [50–52]. Innovative treatment strategies must therefore aim to specifically target these persistent tumor cells. The transcription factor OCT4 has a role in cancer maintenance and resistance to anticancer therapy, thus its expression was studied to characterize persistent cells [5]. Our group previously reported that the OCT4^+^ cell fraction increases after chemotherapy with doxorubicin and targeted therapy with sorafenib or pazopanib in several sarcoma histotypes [9]. Moreover, we studied the antitumor activity of the alkylating agents trabectedin and the PARP1 inhibitor olaparib, observing variable degrees of synergism against advanced sarcomas in preclinical and clinical settings [10, 11]. However, half of the treated patients did not benefit from the combination, and progression or relapse occurred. In this study, we evaluated the phenotype of persistent cells that survived trabectedin and olaparib treatment across a panel of sarcoma histotypes (leiomyosarcoma, chondrosarcoma, fibrosarcoma, undifferentiated pleomorphic sarcoma, and liposarcoma). We focused on monitoring the expression of the OCT4 marker and investigated the molecular mechanisms underlying its regulation, showing that treatment with trabectedin, but not olaparib, significantly increased OCT4 expression in surviving cells. The enrichment of the OCT4⁺ cell fraction suggests that tumor cells persisting after drug treatment acquire cancer stem-like properties, including enhanced resistance to therapy, thereby increasing the risk of disease recurrence [6–8]. Notably, PARP1 plays a central role in maintaining cell pluripotency and stemness-like features, and its inhibition reduces the tumorigenic potential of this treatment-resistant population [13, 53–56]. In line with this, our results showed that olaparib counteracts trabectedin-induced OCT4 upregulation in sarcoma cell lines. To elucidate the molecular mechanism induced by drugs, we took advantage of transcriptomic analysis, which showed that genes involved in *genotoxicity stress* and *cellular response to DNA damage* were significantly modulated by the combination of trabectedin and olaparib. Previous studies have suggested the implications of trabectedin and its derivatives in genotoxicity stress, transcription-dependent replication stress, and genome instability [57–59]. Moreover, by directly blocking PARP1, olaparib impairs the DNA damage response [60] and, in combination with trabectedin, can synergistically perpetuate DNA damage [10]. The global modulation of 413 genes induced by the combination, but not by single agents, confirmed the effective drug synergism in these cell lines. Here, we showed that the perpetuation of DNA damage leads to the accumulation of cytosolic dsDNA, triggering the cGAS-STING pathway, activating the transcription factor IRF3, and eventually promoting IFNβ production in surviving sarcoma cells. Direct IFNβ treatment activates the cGAS–STING pathway, unveiling a feedforward molecular mechanism that enhances OCT4 expression. This finding supports previous evidence of the cancer-promoting effects of type I interferons [23, 61–63]. We showed that IRF3 specifically binds to the *POU5F1* promoter region and directly stimulates gene transcription in response to cGAS-STING pathway activation in sarcoma cells. The upregulation of OCT4 expression promotes cancer stemness and poses a risk for long-term relapse. In addition, we confirmed the involvement of PARP1 in the control of cancer stemness. Notably, cells overexpressing PARP1 exhibit higher levels of OCT4 compared to their parental counterparts. Conversely, PARP1 silencing via shRNA or inhibiting its enzymatic activity with olaparib counteracts the OCT4 upregulation induced by cGAS–STING pathway activation. The apparent contradiction of the PARP1 role in controlling OCT4 may be explained by its non-canonical, transcriptional regulatory functions, beyond its classical role in DNA repair. Several studies have highlighted that PARP1 influences gene transcription by poly-ADP-ribosylating or interacting with transcription factors, chromatin remodelers, and directly RNA polymerase II [64–66]. Focusing on *POU5F1* regulation, it has been shown that PARP1 directly binds to the epigenetic factor PHF20 at the promoter region of *POU5F1,* activating its transcription in neuroblastoma cells [67]. Moreover, the activation of the cGAS-STING pathway in sarcoma cells spared by the trabectedin-olaparib combination upregulates the NK/CIK activating ligand expression, as shown by functional assays after IRF3 silencing or direct IFNβ treatment, adding further insights into the complex regulation of the crosstalk between innate immunity and cancer [20–22, 68]. Future studies analyzing tumor samples from trabectedin-olaparib treated sarcoma patients will provide deeper insights into the evoked molecular mechanisms, particularly regarding the balance between sustaining cancer stemness and immunomodulating effects. Moreover, the results of the exploratory translational analyses from our ongoing phase II clinical trial (NCT03838744) will be instrumental in validating an immunomodulatory signature to help identify patients most likely to benefit from the proposed sequential adoptive cell therapy strategy.

Herein, we demonstrated, for the first time, the enhanced antitumor activity of NK and CIK cells against sarcoma cells pretreated with the trabectedin-olaparib combination, as well as the loss of this efficacy when the cGAS-STING-IRF3-IFNβ pathway is impaired. Notably, we showed that NK and CIK cells effectively targeted and killed both bulk tumor cells and persistent OCT4^+^ cell populations. Therefore, our findings endorse the use of NK/CIK-based immunotherapy to target sarcoma cells that survive drug treatments, consistent with prior evidence from other cancer types [69–71]. These promising results support further investigation of a therapeutic strategy with trabectedin-olaparib combination, followed by sequential treatment with NK/CIK lymphocytes. In vivo preclinical studies will be essential to further support the applicability of this strategy. Xenograft models in immunodeficient mice provide a useful platform to evaluate the direct antitumor effects of adoptive NK/CIK cells. However, the lack of a fully humanized immune system and an intact tumor microenvironment limits the ability to assess potential interactions between adoptive immune cells and host immune or stromal components. Therefore, immunocompetent in vivo preclinical models and ultimately, dedicated clinical trials will be necessary to fully elucidate the complex crosstalk between adoptive NK/CIK cells and the patient’s immune microenvironment.

**Fig. 8:**
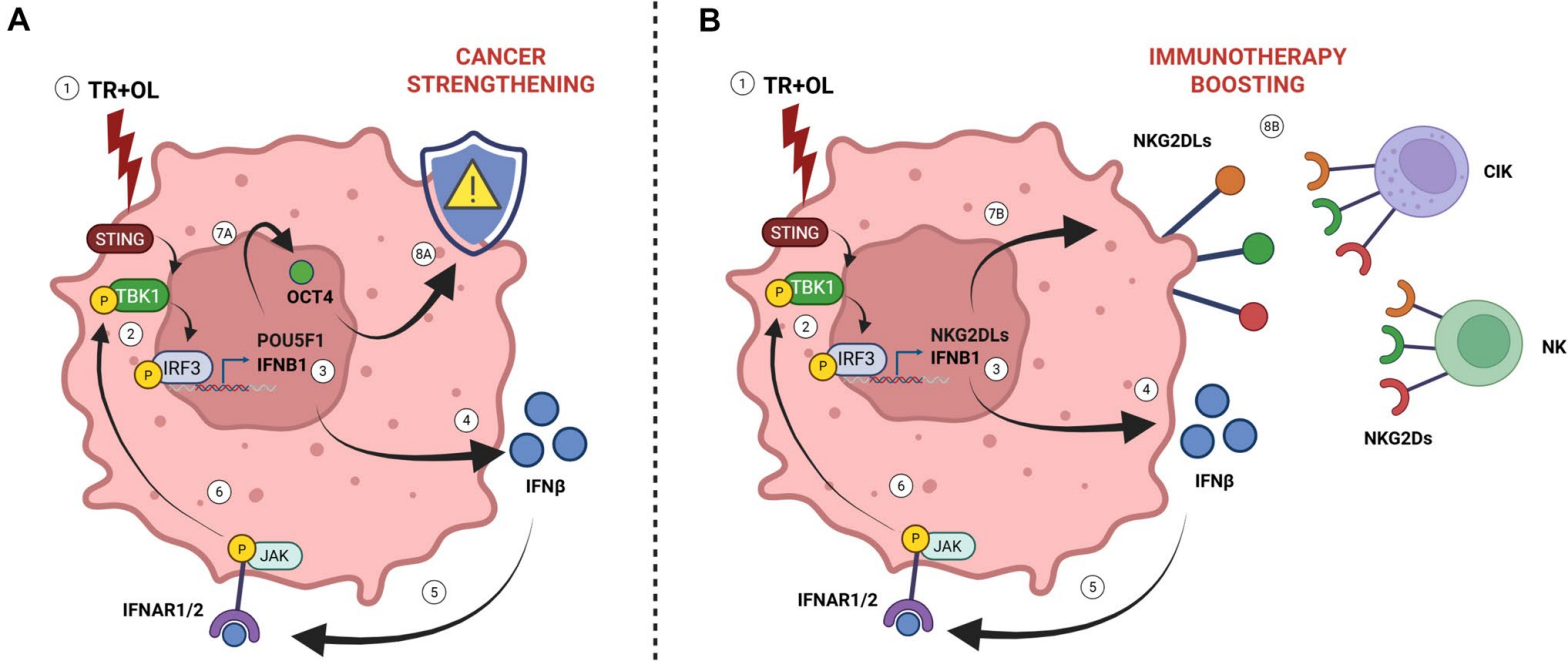
Summary of the molecular mechanism underneath the cGAS-STING pathway modulation of OCT4 and NKG2L expression. The direct treatment with trabectedin-olaparib (1) activates the cGAS-STING pathway through TBK1 and IRF3 phosphorylation (2), leading to *IFNB1* mRNA expression (3) and IFNβ secretion (4). The binding of IFNβ to IFNAR1/2 receptor (5) activates its downstream signaling and the feedforward activation of the cGAS-STING pathway (6). As a double-edged effect, it causes **A** the upregulation of OCT4 (7A) that contributes to cancer strengthening (8A) and, simultaneously, **B** the pro-therapeutic upregulation of NKG2DLs (7B), boosting the NK/CIK immunotherapy (8B)

In conclusion, activation of the cGAS-STING pathway promotes the enrichment of OCT4^+^ persistent cells while simultaneously increasing the expression of NK/CIK activating ligands (Fig. 8). This highlights the dual-edged nature of cGAS-STING pathway modulation, underscoring the need for careful therapeutic targeting to balance the beneficial triggering of anti-tumor immunity and potentially detrimental pro-tumorigenic effects. However, the effective NK/CIK-mediated killing of both the bulk and OCT4 + persistent cells following trabectedin-olaparib treatment, supports the therapeutic potential of this innovative sequential chemo–targeted–immune approach in advanced sarcomas and warrants further investigation.

## Fundings

This work was supported by the Italian Ministry of Health, Ricerca Corrente 2025 and Ricerca Finalizzata - Giovani Ricercatori (GR-2016-02362726 to YP); AIRC (IG 23104 to GGr and IG 20259 to DS), Fondazione per la ricerca sui tumori dell'apparato muscoloscheletrico e rari ONLUS CRT RF = 2016-0917 to GGr; Alleanza Contro il Cancro - working group Sarcomi, Ricerca Corrente - Reti 2021 RCR 2021 WP8 to YP; FPRC 5x1000 Ministero della Salute 2015 - Cancer Im-Gen, FPRC 5x1000 MIUR 2014 - GIST-CRC to GGr and DS; FC was supported by AIRC fellowship “Volontari Comitato Abruzzo-Molise” Rif. 21173; GGi was supported by AIRC Italy Post-Doc ID 31439-2024; AMe research activity was supported by PON 2014-2020 DM 1062/2021 PNR 2021-2027 and PharmaMar (Institutional Grant).

## Supplementary Information

Below is the link to the electronic supplementary material.

Supplementary file1 (DOCX 21 KB)

Supplementary file2 (XLSX 8175 KB)

Supplementary file3 (XLSX 207 KB)

Supplementary file4 (TIFF 46827 KB)

Supplementary file5 (PNG 801 KB)

Supplementary file6 (TIFF 9165 KB)

Supplementary file7 (TIFF 15078 KB)

Supplementary file8 (TIFF 43736 KB)

Supplementary file9 (TIFF 32458 KB)

Supplementary file10 (TIFF 1593 KB)

## Data Availability

The main data supporting the findings of this study are available in the article and its supplementary information. The raw RNA-Seq data are available on GEO ID: GSE239639 (token for Reviewer qbwbawqgjburxqf), all other raw data generated during the study are available from the corresponding authors upon reasonable request.

## Abbreviations

ADP: Adenosine diphosphate
AMP: Adenosine monophosphate
ATCC: American type culture collection
CD: Cluster differentiation
cGAS: Cyclic GMP–AMP synthase
ChIP: Chromatin immunoprecipitation
CIK: Cytokine-induced killer
DMEM: Dulbecco’s modified eagle medium
DMSO: Dimethyl sulfoxide
dsDNA: Double-stranded deoxyribonucleic acid
EMEM: Eagle’s minimum essential medium
FBS: Fetal bovine serum
GMP: Guanosine monophosphate
HLA: Human leukocyte antigen
IFNβ: Interferon-β
IgG: Immunoglobulin G
IL: Interleukine
IRF3: Interferon-regulatory factor 3
MHC: Major histocompatibility complex
MICA/B: MHC class I chain-related proteins A and B
mRNA: Messenger RNA
NK: Natural killer
NKG2DLs: Natural killer group 2D ligands
NOD/SCID: Nonobese diabetic/severe combined immunodeficient mice
OCT4: Octamer-binding transcription factor 4
PARP1: Poly [ADP-ribose] polymerase 1
PBMCs: Peripheral blood mononuclear cells
RPMI: Roswell park memorial institute medium
RT-qPCR: Quantitative reverse transcription polymerase chain reaction
siCTRL: Silencing control
siIRF3: Silencing IRF3
STING: Stimulator of interferon genes
TANK: TRAF family member-associated nuclear factor-kappa-B activator
TBK1: TANK binding kinase-1
TRAF: Tumor necrosis factor receptor-associated factor
ULBP: UL16 binding protein

## References

[1] Blay JY, Honoré C, Stoeckle E, Meeus P, Jafari M, Gouin F et al (2019) Surgery in reference centers improves survival of sarcoma patients: a nationwide study. Ann Oncol 30:1143–1153 31081028 10.1093/annonc/mdz124 PMC6637376

[2] Shen S, Vagner S, Robert C (2020) Persistent cancer cells: the deadly survivors. Cell 183:860–874 33186528 10.1016/j.cell.2020.10.027

[3] Clara JA, Monge C, Yang Y, Takebe N (2020) Targeting signalling pathways and the immune microenvironment of cancer stem cells—a clinical update. Nat Rev Clin Oncol 17:204–232 31792354 10.1038/s41571-019-0293-2

[4] Rosner MH, Vigano MA, Ozato K, Timmons PM, Poirier F, Rigby PW et al (1990) A POU-domain transcription factor in early stem cells and germ cells of the mammalian embryo. Nature 345:686–692 1972777 10.1038/345686a0

[5] Hu T, Liu S, Breiter DR, Wang F, Tang Y, Sun S (2008) Octamer 4 small interfering RNA results in cancer stem cell-like cell apoptosis. Cancer Res 68:6533–6540 18701476 10.1158/0008-5472.CAN-07-6642

[6] Zhou HM, Zhang JG, Zhang X, Lin Q (2021) Targeting cancer stem cells for reversing therapy resistance: mechanism, signaling, and prospective agents. Sig Transduct Target Ther 6:62 10.1038/s41392-020-00430-1 PMC7884707 33589595

[7] Lu CS, Shieh GS, Wang CT, Su BH, Su YC, Chen YC et al (2017) Chemotherapeutics-induced Oct4 expression contributes to drug resistance and tumor recurrence in bladder cancer. Oncotarget 8:30844–30858 27244887 10.18632/oncotarget.9602 PMC5458172

[8] Dong Z, Zeng Q, Luo H, Zou J, Cao C, Liang J et al (2012) Increased expression of OCT4 is associated with low differentiation and tumor recurrence in human hepatocellular carcinoma. Pathol Res Pract 208:527–533 22824146 10.1016/j.prp.2012.05.019

[9] Mesiano G, Grignani G, Fiorino E, Leuci V, Rotolo R, D’Ambrosio L et al (2018) Cytokine induced killer cells are effective against sarcoma cancer stem cells spared by chemotherapy and target therapy. Oncoimmunology 7:1–11 10.1080/2162402X.2018.1465161 PMC6208452 30393581

[10] Pignochino Y, Capozzi F, D’Ambrosio L, Dell’Aglio C, Basiricò M, Canta M et al (2017) PARP1 expression drives the synergistic antitumor activity of trabectedin and PARP1 inhibitors in sarcoma preclinical models. Mol Cancer 16:86 28454547 10.1186/s12943-017-0652-5 PMC5410089

[11] Grignani G, Ambrosio LD, Pignochino Y, Palmerini E, Zucchetti M, Boccone P et al (2018) Trabectedin and olaparib in patients with advanced and non-resectable bone and soft-tissue sarcomas (TOMAS): an open-label, phase 1b study from the Italian sarcoma Group. Lancet Oncol 19:1360–1371 30217671 10.1016/S1470-2045(18)30438-8

[12] Martinez-Cruzado L, Tornin J, Rodriguez A, Santos L, Allonca E, Fernandez-Garcia MT et al (2017) Trabectedin and camptothecin synergistically eliminate cancer stem cells in cell-of-origin sarcoma models. Neoplasia 19:460–470 28494349 10.1016/j.neo.2017.03.004 PMC5421973

[13] Shimo T, Kurebayashi J, Kanomata N, Yamashita T, Kozuka Y, Moriya T et al (2014) Antitumor and anticancer stem cell activity of a poly ADP-ribose polymerase inhibitor olaparib in breast cancer cells. Breast Cancer 21:75–85 22454224 10.1007/s12282-012-0356-z

[14] Yang XD, Kong FE, Qi L, Lin JX, Yan Q, Loong JHC et al (2021) PARP inhibitor olaparib overcomes sorafenib resistance through reshaping the pluripotent transcriptome in hepatocellular carcinoma. Mol Cancer 20:20 33485358 10.1186/s12943-021-01315-9 PMC7824946

[15] Bellio C, DiGloria C, Foster R, James K, Konstantinopoulos PA, Growdon WB et al (2019) PARP inhibition induces enrichment of DNA repair-proficient CD133 and CD117 positive ovarian cancer stem cells. Mol Cancer Res 17:431–445 30401718 10.1158/1541-7786.MCR-18-0594

[16] Shen J, Zhao W, Ju Z, Wang L, Peng Y, Labrie M et al (2019) PARpi triggers the STING-dependent immune response and enhances the therapeutic efficacy of immune checkpoint blockade independent of BRCAness. Cancer Res 79:311–319 30482774 10.1158/0008-5472.CAN-18-1003 PMC6588002

[17] Kim C, Wang XD, Yu Y (2020) PARP1 inhibitors trigger innate immunity via PARP1 trapping-induced DNA damage response. Elife 9:e60637 32844745 10.7554/eLife.60637 PMC7486119

[18] Wang F, Zhao M, Chang B, Zhou Y, Wu X, Ma M et al (2022) Cytoplasmic PARP1 links genome instability to the inhibition of antiviral immunity through PARylating cGAS. Mol Cell 82(11):2032–2049 35460603 10.1016/j.molcel.2022.03.034

[19] Salaroglio IC, Aviles P, Kopecka J et al (2024) Ecteinascidin synthetic analogues: a new class of selective inhibitors of transcription, exerting immunogenic cell death in refractory malignant pleural mesothelioma. J Exp Clin Cancer Res 43:327 39709435 10.1186/s13046-024-03253-y PMC11662834

[20] Gasser S, Orsulic S, Brown EJ, Raulet DH (2005) The DNA damage pathway regulates innate immune system ligands of the NKG2D receptor. Nature 436:1186–1190 15995699 10.1038/nature03884 PMC1352168

[21] Lam AR, Bert NL, Ho SS, Shen YJ, Tang LF, Xiong GM et al (2014) RAE1 ligands for the NKG2D receptor are regulated by STING-dependent DNA sensor pathways in lymphoma. Cancer Res 74:2193–2203 24590060 10.1158/0008-5472.CAN-13-1703 PMC4229084

[22] Woo SR, Fuertes MB, Corrales L, Spranger S, Furdyna MJ, Leung MY, Duggan R, Wang Y, Barber GN, Fitzgerald KA, Alegre ML, Gajewski TF (2014) STING-dependent cytosolic DNA sensing mediates innate immune recognition of immunogenic tumors. Immunity 41:830–4243 25517615 10.1016/j.immuni.2014.10.017 PMC4384884

[23] Liu FR, Jiang MJ, Mei Z, Lin CJ, Tian L (2022) cGAS-STING signalings potentiate tumor progression via sustaining cancer stemness. Transl Oncol 20:101404 35364558 10.1016/j.tranon.2022.101404 PMC8968062

[24] Hong C, Schubert M, Tijhuis AE, Requesens M, Roorda M, van den Brink A et al (2022) cGAS–STING drives the IL-6-dependent survival of chromosomally instable cancers. Nature 607:366–373 35705809 10.1038/s41586-022-04847-2

[25] Yi SY, Wei MZ, Zhao L (2024) Targeted immunotherapy to cancer stem cells: a novel strategy of anticancer immunotherapy. Crit Rev Oncol Hematol 196:104313 38428702 10.1016/j.critrevonc.2024.104313

[26] Laskowski TJ, Biederstädt A, Rezvani K (2022) Natural killer cells in antitumour adoptive cell immunotherapy. Nat Rev Cancer 22:557–575 35879429 10.1038/s41568-022-00491-0 PMC9309992

[27] Wolf NK, Kissiov DU, Raulet DH (2023) Roles of natural killer cells in immunity to cancer, and applications to immunotherapy. Nat Rev Immunol 23:90–105 35637393 10.1038/s41577-022-00732-1

[28] Mesiano G, Leuci V, Giraudo L, Gammaitoni L, Carnevale Schianca F, Cangemi M et al (2015) Adoptive immunotherapy against sarcomas. Expert Opin Biol Ther 15:517–528 25516119 10.1517/14712598.2015.987121

[29] Sangiolo D, Mesiano G, Gammaitoni L, Leuci V, Todorovic M, Giraudo L et al (2014) Cytokine-induced killer cells eradicate bone and soft-tissue sarcomas. Cancer Res 74:119–129 24356422 10.1158/0008-5472.CAN-13-1559

[30] Bauer S, Groh V, Wu J, Steinle A, Phillips JH, Lanier LL et al (1999) Activation of NK cells and T cells by NKG2D, a receptor for stress-inducible MICA. Science 285:727–729 10426993 10.1126/science.285.5428.727

[31] Nausch N, Cerwenka A (2008) NKG2D ligands in tumor immunity. Oncogene 27:5944–5958 18836475 10.1038/onc.2008.272

[32] Sayitoglu EC, Georgoudaki AM, Chrobok M, Ozkazanc D, Josey BJ, Arif M et al (2020) Boosting natural killer cell-mediated targeting of sarcoma through DNAM-1 and NKG2D. Front Immunol 11:1–20 32082316 10.3389/fimmu.2020.00040 PMC7001093

[33] Cucè M, Gallo Cantafio ME, Siciliano MA, Riillo C, Caracciolo D, Scionti F et al (2019) Trabectedin triggers direct and NK-mediated cytotoxicity in multiple myeloma. J Hematol Oncol 12:1–17 30898137 10.1186/s13045-019-0714-9 PMC6429746

[34] Soriani A, Zingoni A, Cerboni C, Lannitto ML, Ricciardi MR, Di GV et al (2009) ATM-ATR-dependent up-regulation of DNAM-1 and NKG2D ligands on multiple myeloma cells by therapeutic agents results in enhanced NK-cell susceptibility and is associated with a senescent phenotype. Blood 113:3503–3511 19098271 10.1182/blood-2008-08-173914

[35] Peterse EFP, Niessen B, Addie RD, de Jong Y, Cleven AHG, Kruisselbrink AB et al (2018) Targeting glutaminolysis in chondrosarcoma in context of the IDH1/2 mutation. Br J Cancer 118:1074–1083 29576625 10.1038/s41416-018-0050-9 PMC5931088

[36] Li L, Paz AC, Wilky BA, Johnson B, Galoian K, Rosenberg A et al (2015) Treatment with a small molecule mutant IDH1 inhibitor suppresses tumorigenic activity and decreases production of the oncometabolite 2-hydroxyglutarate in human chondrosarcoma cells. PLoS ONE 10:e0133813 26368816 10.1371/journal.pone.0133813 PMC4569544

[37] Chiabotto G, Grignani G, Todorovic M, Martin V, Centomo ML, Prola E et al (2020) Pazopanib and trametinib as a synergistic strategy against osteosarcoma: preclinical activity and molecular insights. Cancers 12:1–21 10.3390/cancers12061519 PMC7352822 32531992

[38] Chen S, Zhou Y, Chen Y, Gu J (2018) Fastp: An ultra-fast all-in-one FASTQ preprocessor. Bioinformatics 34:i884–90 30423086 10.1093/bioinformatics/bty560 PMC6129281

[39] Patro R, Duggal G, Love MI, Irizarry RA, Kingsford C (2017) Salmon provides fast and bias-aware quantification of transcript expression. Nat Methods 14:417–419 28263959 10.1038/nmeth.4197 PMC5600148

[40] Soneson C, Love MI, Robinson MD (2015) Differential analyses for RNA-seq: transcript-level estimates improve gene-level inferences. F1000Res 4:1521 26925227 10.12688/f1000research.7563.1 PMC4712774

[41] Love MI, Huber W, Anders S (2014) Moderated estimation of fold change and dispersion for RNA-seq data with DESeq2. Genome Biol 15:1–21 10.1186/s13059-014-0550-8 PMC4302049 25516281

[42] Zhou Y, Zhou B, Pache L, Chang M, Khodabakhshi AH, Tanaseichuk O et al (2019) Metascape provides a biologist-oriented resource for the analysis of systems-level datasets. Nat Commun 10:1523 30944313 10.1038/s41467-019-09234-6 PMC6447622

[43] Schneider CA, Rasband WS, Eliceiri KW (2012) NIH image to ImageJ: 25 years of image analysis. Nat Methods 9:671–675 22930834 10.1038/nmeth.2089 PMC5554542

[44] Dreos R, Ambrosini G, Groux R, Cavin Périer R, Bucher P (2017) The eukaryotic promoter database in its 30th year: focus on non-vertebrate organisms. Nucleic Acids Res 45:D51–D55 27899657 10.1093/nar/gkw1069 PMC5210552

[45] Bailey TL, Johnson J, Grant CE, Noble WS (2015) The MEME Suite. Nucleic Acids Res 43:W39-49 25953851 10.1093/nar/gkv416 PMC4489269

[46] Rauluseviciute I, Riudavets-Puig R, Blanc-Mathieu R, Castro-Mondragon JA, Ferenc K, Kumar V et al (2024) JASPAR 2024: 20th anniversary of the open-access database of transcription factor binding profiles. Nucleic Acids Res 52:D174–D182 37962376 10.1093/nar/gkad1059 PMC10767809

[47] Chen Q, Sun L, Chen ZJ (2016) Regulation and function of the cGAS-STING pathway of cytosolic DNA sensing. Nat Immunol 17:1142–1149 27648547 10.1038/ni.3558

[48] Le Bert N, Lam AR, Ho SS, Shen YJ, Liu MM, Gasser S (2014) STING-dependent cytosolic DNA sensor pathways regulate NKG2D ligand expression. Oncoimmunology 3:e29259 25114832 10.4161/onci.29259 PMC4126837

[49] Lu L, Yang C, Zhou X, Wu L, Hong X, Li W, Wang X, Yang Y, Cao D, Zhang A, Di W, Deng L (2023) STING signaling promotes NK cell antitumor immunity and maintains a reservoir of TCF-1^+^ NK cells. Cell Rep 42:113108 37708030 10.1016/j.celrep.2023.113108

[50] Genadry KC, Pietrobono S, Rota R, Linardic CM (2018) Soft tissue sarcoma cancer stem cells: an overview. Front Oncol 8:1–17 30416982 10.3389/fonc.2018.00475 PMC6212576

[51] Canter RJ, Ames E, Mac S, Grossenbacher SK, Chen M, Li CS et al (2014) Anti-proliferative but not anti-angiogenic tyrosine kinase inhibitors enrich for cancer stem cells in soft tissue sarcoma. BMC Cancer 14:1–13 25301268 10.1186/1471-2407-14-756 PMC4200119

[52] Ames E, Canter RJ, Grossenbacher SK, Mac S, Smith RC, Monjazeb AM et al (2015) Enhanced targeting of stem-like solid tumor cells with radiation and natural killer cells. Oncoimmunology 4:1–11 10.1080/2162402X.2015.1036212 PMC4570100 26405602

[53] Jiang BH, Tseng WL, Li HY, Wang ML, Chang YL, Sung YJ et al (2015) Poly(ADP-ribose) polymerase 1: cellular pluripotency, reprogramming, and tumorogenesis. Int J Mol Sci 16:15531–15545 26184161 10.3390/ijms160715531 PMC4519911

[54] Zeniou M, Nguekeu-Zebaze L, Dantzer F (2019) Therapeutic considerations of PARP in stem cell biology: relevance in cancer and beyond. Biochem Pharmacol 167:107–115 31202733 10.1016/j.bcp.2019.06.012

[55] Quiñonero F, Mesas C, Muñoz-Gámez JA, Jiménez-Luna C, Perazzoli G, Prados J et al (2022) PARP1 inhibition by Olaparib reduces the lethality of pancreatic cancer cells and increases their sensitivity to Gemcitabine. Biomed Pharmacother 155:113669 36113257 10.1016/j.biopha.2022.113669

[56] Puentes-Pardo JD, Moreno-SanJuan S, Casado J, Escudero-Feliu J, López-Pérez D, Sánchez-Uceta P et al (2023) PARP-1 expression influences cancer stem cell phenotype in colorectal cancer depending on p53. Int J Mol Sci 24:4787 36902215 10.3390/ijms24054787 PMC10002521

[57] D’Incalci MD, Badri N, Galmarini CM, Allavena P (2014) Trabectedin, a drug acting on both cancer cells and the tumour microenvironment. Br J Cancer 111:646–650 24755886 10.1038/bjc.2014.149 PMC4134488

[58] Tumini E, Herrera-Moyano E, Martín-Alonso MS, Barroso S, Galmarini CM, Aguilera A (2019) The antitumor drugs trabectedin and lurbinectedin induce transcription-dependent replication stress and genome instability. Mol Cancer Res 17(3):773–782 30552231 10.1158/1541-7786.MCR-18-0575 PMC6398590

[59] Son K, Takhaveev V, Mor V et al (2024) Trabectedin derails transcription-coupled nucleotide excision repair to induce DNA breaks in highly transcribed genes. Nat Commun 15:1388 38360910 10.1038/s41467-024-45664-7 PMC10869700

[60] Grignani G, Merlini A, Sangiolo D, Ambrosio D, Pignochino Y (2020) Delving into PARP inhibition from bench to bedside and back. Pharmacol Ther 206:107446 31756364 10.1016/j.pharmthera.2019.107446

[61] Musella M, Guarracino A, Manduca N, Galassi C, Ruggiero E, Potenza A et al (2022) Type I IFNs promote cancer cell stemness by triggering the epigenetic regulator KDM1B. Nat Immunol 23:1379–1392 36002648 10.1038/s41590-022-01290-3 PMC9477743

[62] De Martino M, Vanpouille-Box C (2022) Type I interferon induces cancer stem cells-mediated chemotherapy resistance. Oncoimmunology 11:2127274 36185803 10.1080/2162402X.2022.2127274 PMC9518997

[63] Tong Z, Zou JP, Wang SY, Luo WW, Wang YY (2024) Activation of the cGAS-STING-IRF3 axis by type I and II interferons contributes to host defense. Adv Sci 11:e2308890 10.1002/advs.202308890 PMC11425201 39004913

[64] Ahel D, Horejsí Z, Wiechens N, Polo SE, Garcia-Wilson E, Ahel I et al (2009) Poly(ADP-ribose)-dependent regulation of DNA repair by the chromatin remodeling enzyme ALC1. Science 325:1240–1243 19661379 10.1126/science.1177321 PMC3443743

[65] Krishnakumar R, Kraus WL (2010) PARP-1 regulates chromatin structure and transcription through a KDM5B-dependent pathway. Mol Cell 39:736–749 20832725 10.1016/j.molcel.2010.08.014 PMC2939044

[66] Gibson BA, Zhang Y, Jiang H, Hussey KM, Shrimp JH, Lin H et al (2016) Chemical genetic discovery of PARP targets reveals a role for PARP-1 in transcription elongation. Science 353:45–50 27256882 10.1126/science.aaf7865 PMC5540732

[67] Long W, Zhao W, Ning B, Huang J, Chu J, Li L, Ma Q, Xing C, Wang HY, Liu Q, Wang RF (2018) PHF20 collaborates with PARP1 to promote stemness and aggressiveness of neuroblastoma cells through activation of SOX2 and OCT4. J Mol Cell Biol 10:147–160 29452418 10.1093/jmcb/mjy007 PMC5951121

[68] Tamura T, Yanai H, Savitsky D, Taniguchi T (2008) The IRF family transcription factors in immunity and oncogenesis. Annu Rev Immunol 26:535–584 18303999 10.1146/annurev.immunol.26.021607.090400

[69] Tallerico R, Todaro M, Di Franco S, Maccalli C, Garofalo C, Sottile R et al (2013) Human NK cells selective targeting of colon cancer-initiating cells: a role for natural cytotoxicity receptors and MHC class I molecules. J Immunol 190:2381–2390 23345327 10.4049/jimmunol.1201542

[70] Castriconi R, Daga A, Dondero A, Zona G, Poliani PL, Melotti A et al (2009) NK cells recognize and kill human glioblastoma cells with stem cell-like properties. J Immunol 182:3530–3539 19265131 10.4049/jimmunol.0802845

[71] Pietra G, Manzini C, Vitale M, Balsamo M, Ognio E, Boitano M et al (2009) Natural killer cells kill human melanoma cells with characteristics of cancer stem cells. Int Immunol 21:793–801 19491215 10.1093/intimm/dxp047

