# Supplementary material for: The cGAS-STING pathway is a master regulator of OCT4 expression in persistent sarcoma cells and enhances cellular immunotherapy with NK and CIK lymphocytes
Source: Cancer Immunol Immunother. 2025 Sep 23;74(10):312. doi: 10.1007/s00262-025-04141-w (PMC12457259; doi:10.1007/s00262-025-04141-w)
Supplement: Supplementary file 1 — Supplementary file1 (DOCX 21 KB) [file 262_2025_4141_MOESM1_ESM.docx]

**Supplementary Table S1**. List of siRNAs employed for gene silencing

| Target Silencer | Company and siRNA ID |  |
| --- | --- | --- |
| IFNB1  IRF3  CTRL | Thermo Fisher Scientific, 144950 and s7187  Thermo Fisher Scientific, 106517  Thermo Fisher Scientific, N#1 |  |

**Supplementary Table S2**. List of TaqMan probes employed for qRT-PCR

| Human TaqMan probes | Companies and Catalog |
| --- | --- |
| MICA  MICB | Thermo Fisher Scientific, Hs00792195_m1  Thermo Fisher Scientific, Hs00792952_m1 |
| ULBP2  ULBP3  POU5F1 (OCT4)  TMEM173 (STING1)  TBK1  IRF3  IFNB1  ACTB | Thermo Fisher Scientific, Hs01127964_m1  Thermo Fisher Scientific, Hs00225909_m1  Thermo Fisher Scientific, Hs04260367_gH  Thermo Fisher Scientific, Hs00736955_g1  Thermo Fisher Scientific, Hs00179410_m1  Thermo Fisher Scientific, Hs00155574_m1  Thermo Fisher Scientific, Hs01077958_s1  Thermo Fisher Scientific, Hs03023943_g1 |

**Supplementary Table S3.** List of primers employed for gene expression analysis by RT-qPCR or q-PCR

| Human genes | Primer sequences |
| --- | --- |
| IFNAR1 | Forward 5’-AGCCCCACTAAACAAAGCAC -3’  Reverse 5’- GCAATCCCCTCAAAGACTGA -3’ |
| IFNAR2 | Forward 5’- CTCACAGTTTGTAAATCTTTTTCCC -3’  Reverse 5’- AGGGAGAAGTGAAAGTGGGAA -3’ |
| GUSB | Forward 5’- CGCTATGGG ATTGTGGTCAT-3’  Reverse 5’- CATGTGGTGATGCAGAGAAA-3’ |

**Supplementary Table S4**. List of antibodies employed for western blot, ChIP, and immunofluorescence

| Antibodies | Company and Catalog number | Application |
| --- | --- | --- |
| OCT4  IFNAR1  TBK1 | Proteintech, 11263-1-AP  Proteintech, 13083-1-AP  Thermo Fisher Scientific, PA517478 | Western blot  Western blot  Western blot |
| P-TBK1 (Ser172)  IRF3  P-IRF3 (Ser396)  MICA  MICB  ULBP2  ULBP3  STAT1  P-STAT1(Tyr701)  β-ACTIN  VINCULIN  IRF3  IgG  dsDNA | Thermo Fisher Scientific, PA5105919  Thermo Fisher Scientific, PA520087  Thermo Fisher Scientific, #720012  Thermo Fisher Scientific, PA535346  Thermo Fisher Scientific, PA578061  Thermo Fisher Scientific, MA538656  Abcam, ab259988  Cell Signaling Technology, #9172  Cell Signaling Technology, #9167  Cell Signaling Technology, #4970  Cell Signaling Technology, #13901  Cell Signaling Technology, 4302S  Diagenode, C15400001-15  Abcam, #ab27156 | Western blot  Western blot  Western blot  Western blot  Western blot  Western blot  Western blot  Western blot  Western blot  Western blot  Western blot  ChIP  ChIP  Immunofluorescence |

**Supplementary Table S5**. List of antibodies employed for flow cytometry

| Target | Conjugate | Companies and Catalog |
| --- | --- | --- |
| MICA/B  ULBP2/5/6 | PE  APC | BD Pharmingen, 558352  R&D System, FAB1298A |
| ULBP3  CD3  CD8  CD56  CD134 (NKG2D)  CD226 (DNAM1)  OCT4 | PE  FITC  PE  APC  APC  PE  PE | R&D System, FAB1517P  Miltenyi Biotech,130-120-267  Miltenyi Biotech, 130-110-678  Miltenyi Biotech, 130-113-305  Miltenyi Biotech, 130-098-844  BD Pharmingen, 567358  Miltenyi Biotech, REA338 |

**Supplementary Table S6.** List of primers employed for promoter analysis by ChIP-qPCR

| Human genes | Primer sequences |
| --- | --- |
| POU5F1 | Forward 5’- AGCCCCACTAAACAAAGCAC -3’  Reverse 5’- GCAATCCCCTCAAAGACTGA-3’ |
| IFNB1 | Forward 5’-CTCACAGTTTGTAAATCTTTTTCCC-3’  Reverse 5’- TTCCCACTTTCACTTCTCCCT -3’ |

**Supplementary Table S7**. **List of all and differentially expressed genes from RNA-Seq data analysis.** Median expression, fold increase (log2FC), p value, and adjusted p-value of genes with median expression >1, as calculated by bioinformatic analysis of RNA-Seq data from treated cell lines and xenografts.

**Supplementary Table S8**. **Metascape and Gene Set Enrichment Analysis.** The first sheet contains the annotations for the analyzed DE genes, while the second, third, and fourth sheets display the enriched terms for all DE genes, and separately for those that are upregulated or downregulated by the treatments. The fifth sheet contains the results of the GSEA analysis, showing p-value and NES.
