# Supplementary figures and images for: The cGAS-STING pathway is a master regulator of OCT4 expression in persistent sarcoma cells and enhances cellular immunotherapy with NK and CIK lymphocytes
Source: Cancer Immunol Immunother. 2025 Sep 23;74(10):312. doi: 10.1007/s00262-025-04141-w (PMC12457259; doi:10.1007/s00262-025-04141-w)

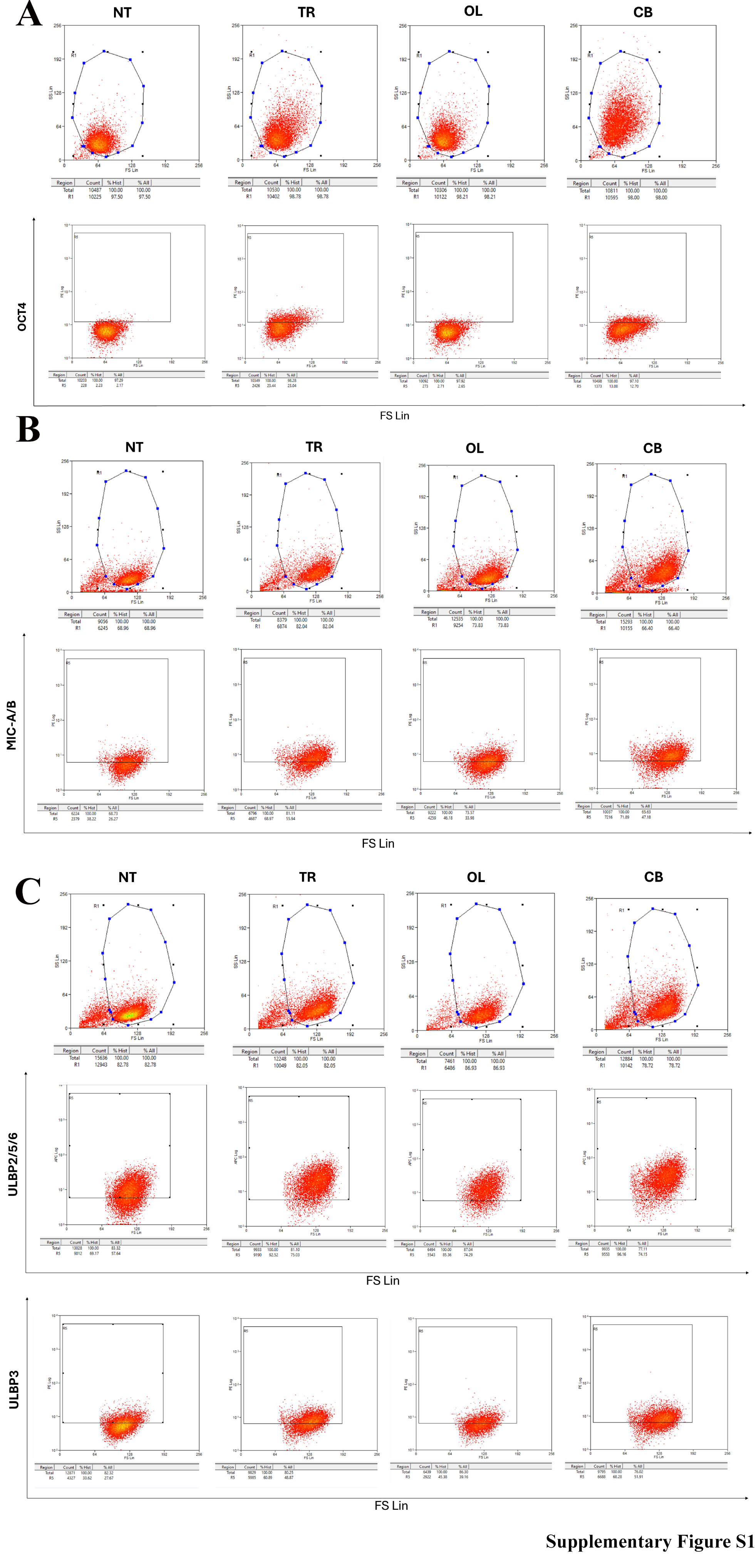

Supplement: Supplementary file 4 — Supplementary file4 (TIFF 46827 KB) [file 262_2025_4141_MOESM4_ESM.tif]

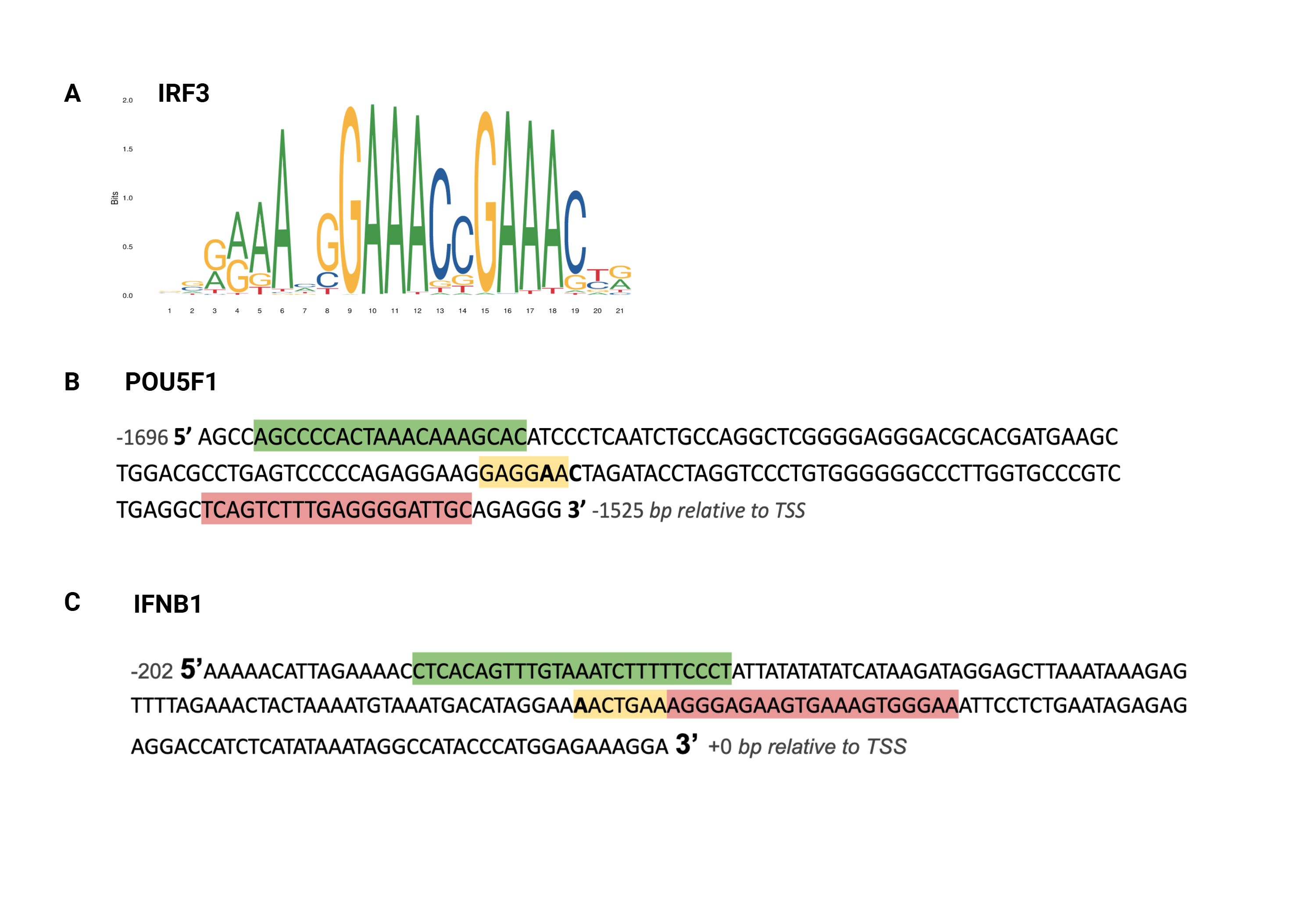

Supplement: Supplementary file 5 — Supplementary file5 (PNG 801 KB) [file 262_2025_4141_MOESM5_ESM.png]

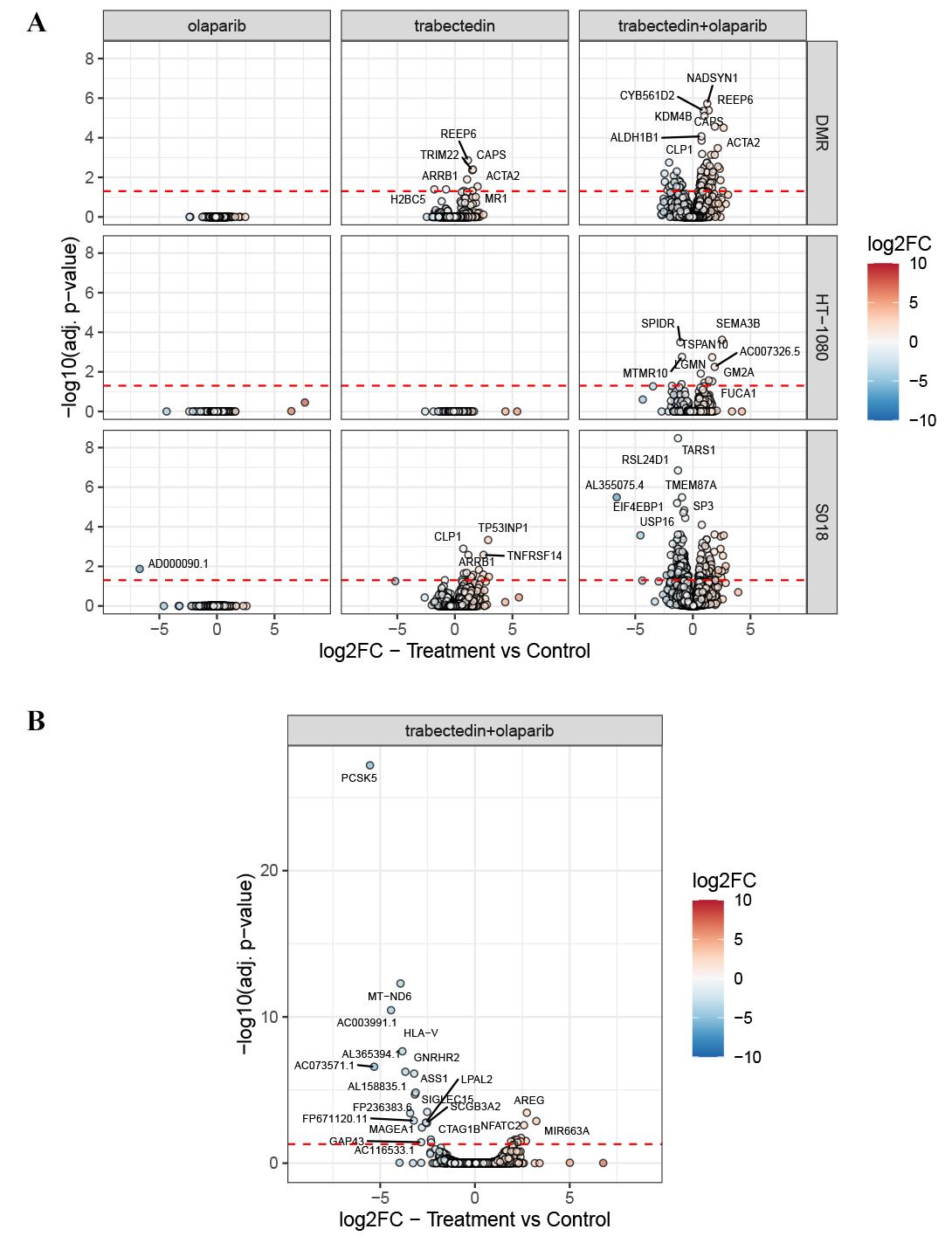

Supplement: Supplementary file 6 — Supplementary file6 (TIFF 9165 KB) [file 262_2025_4141_MOESM6_ESM.tif]

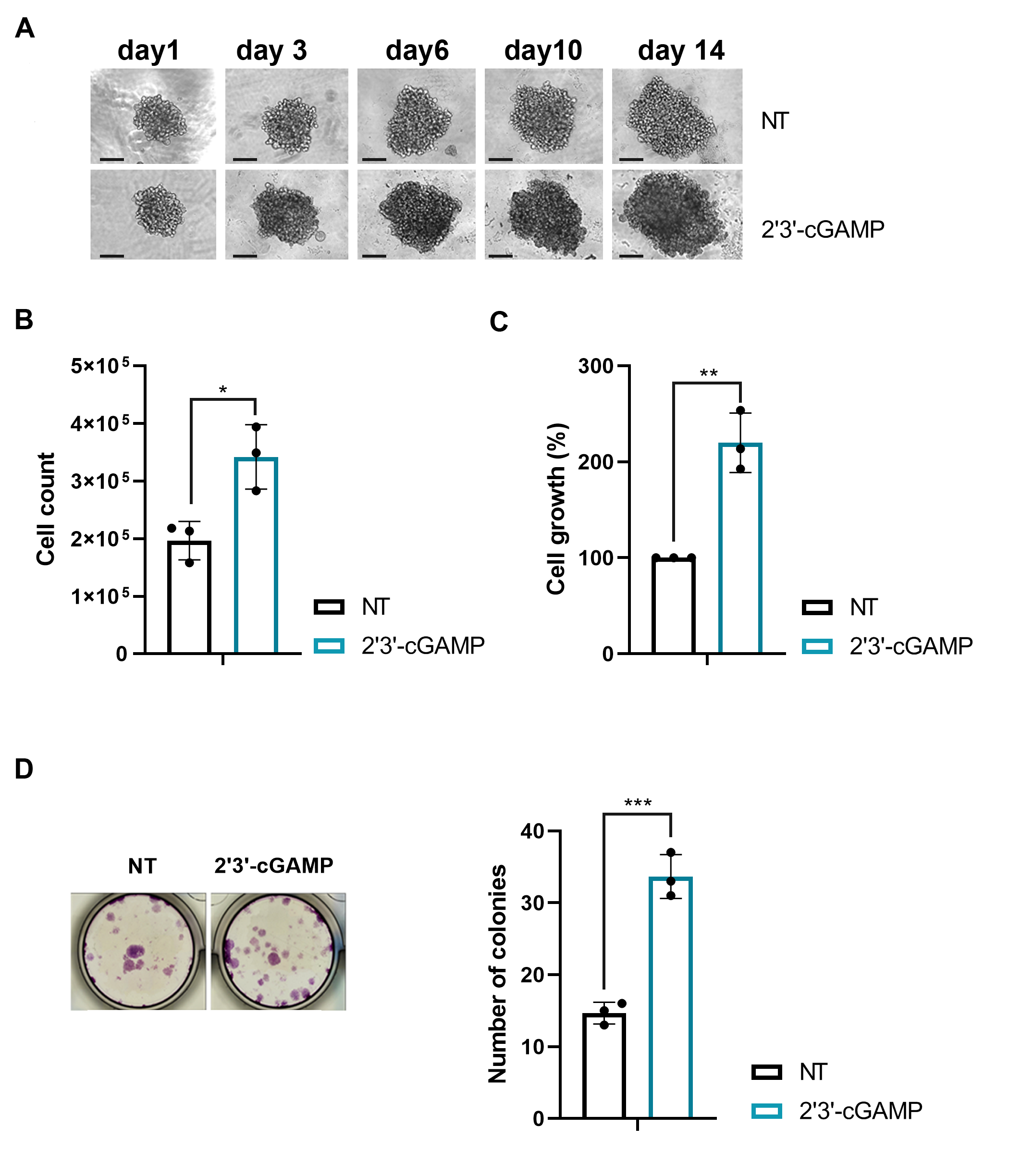

Supplement: Supplementary file 7 — Supplementary file7 (TIFF 15078 KB) [file 262_2025_4141_MOESM7_ESM.tif]

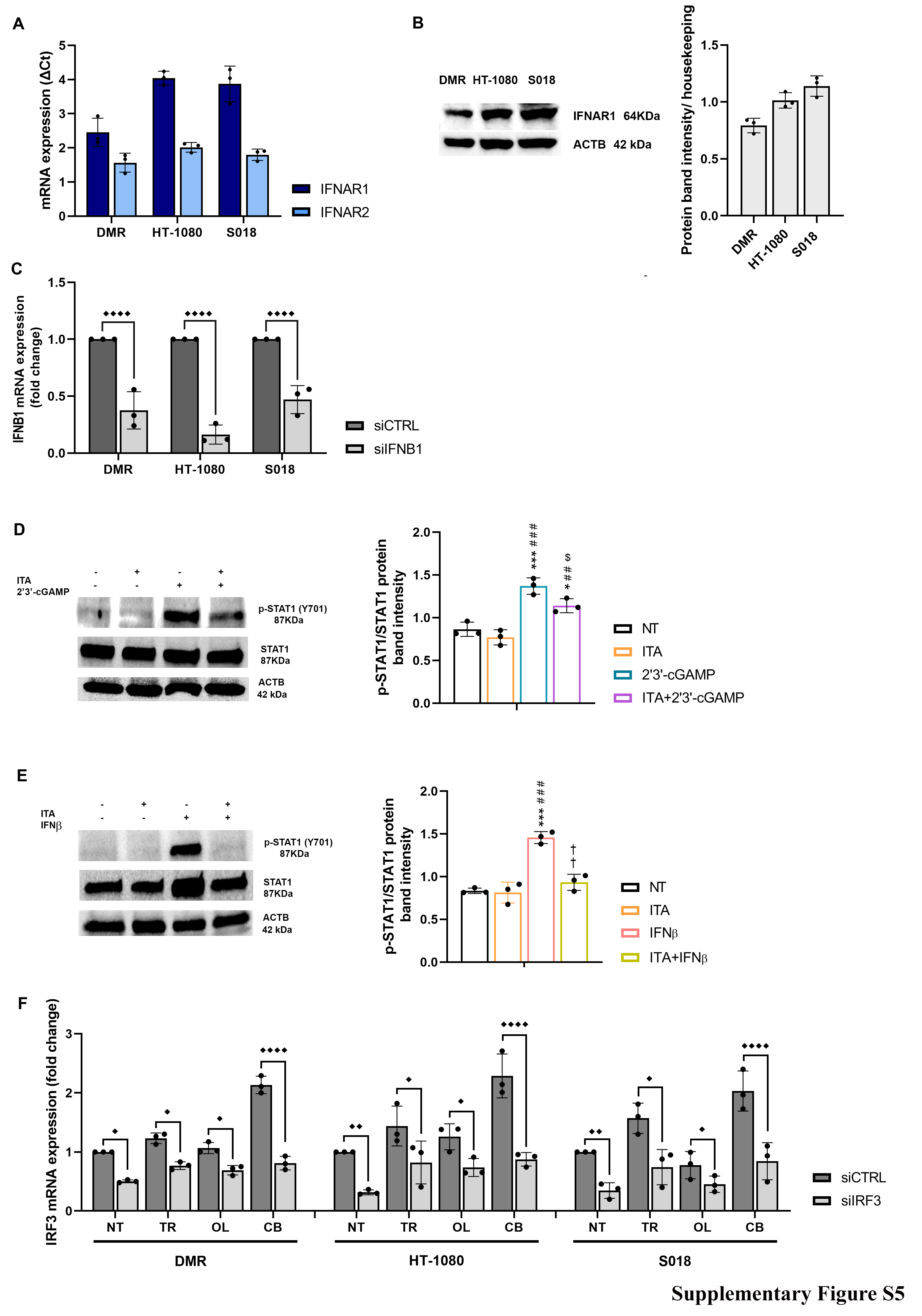

Supplement: Supplementary file 8 — Supplementary file8 (TIFF 43736 KB) [file 262_2025_4141_MOESM8_ESM.tif]

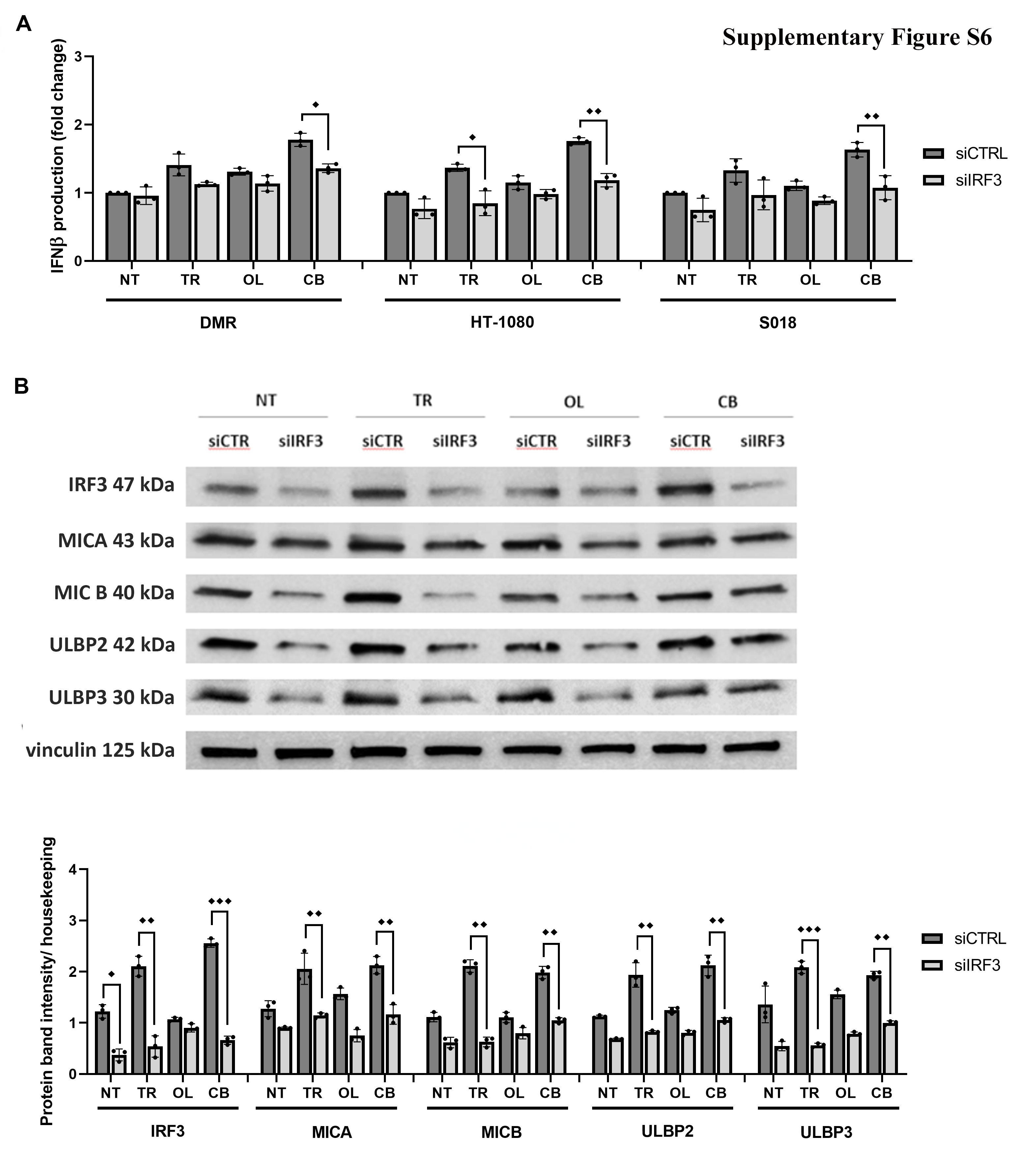

Supplement: Supplementary file 9 — Supplementary file9 (TIFF 32458 KB) [file 262_2025_4141_MOESM9_ESM.tif]

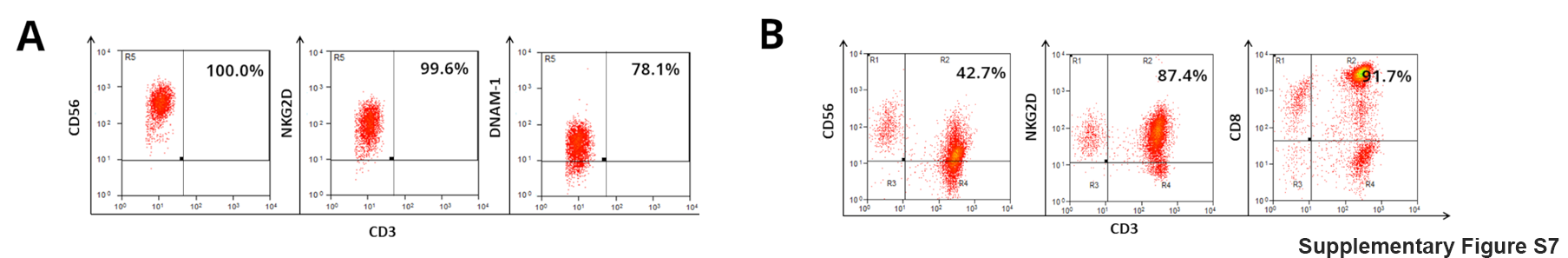

Supplement: Supplementary file 10 — Supplementary file10 (TIFF 1593 KB) [file 262_2025_4141_MOESM10_ESM.tif]
